# Colocalization of Cancer-Associated Biomarkers on Single Extracellular Vesicles for Early Detection of Cancer

**DOI:** 10.1016/j.jmoldx.2024.08.006

**Published:** 2024-09-24

**Authors:** Daniel P. Salem, Laura T. Bortolin, Dan Gusenleitner, Jonian Grosha, Ibukunoluwapo O. Zabroski, Kelly M. Biette, Sanchari Banerjee, Christopher R. Sedlak, Delaney M. Byrne, Bilal F. Hamzeh, MacKenzie S. King, Lauren T. Cuoco, Timothy Santos-Heiman, Gabrielle N. Barcaskey, Katherine S. Yang, Peter A. Duff, Emily S. Winn-Deen, Toumy Guettouche, Dawn R. Mattoon, Eric K. Huang, Randy W. Schekman, Anthony D. Couvillon, Joseph C. Sedlak

**Affiliations:** ∗ Mercy BioAnalytics Inc., Waltham, Massachusetts; † Department of Molecular and Cell Biology, Li Ka Shing Center, University of California Berkeley, Berkeley, California

## Abstract

Detection of cancer early, when it is most treatable, remains a significant challenge because of the lack of diagnostic methods sufficiently sensitive to detect nascent tumors. Early-stage tumors are small relative to their tissue of origin, heterogeneous, and infrequently manifest in clinical symptoms. The detection of early-stage tumors is challenging given the lack of tumor-specific indicators (ie, protein biomarkers, circulating tumor DNA) to enable detection using a noninvasive diagnostic assay. To overcome these obstacles, we have developed a liquid biopsy assay that interrogates circulating extracellular vesicles (EVs) to detect tumor-specific biomarkers colocalized on the surface of individual EVs. We demonstrate the technical feasibility of this approach in human cancer cell line–derived EVs, where we show strong correlations between assay signal and cell line gene/protein expression for the ovarian cancer–associated biomarkers bone marrow stromal antigen-2, folate receptor-α, and mucin-1. Furthermore, we demonstrate that detecting distinct colocalized biomarkers on the surface of EVs significantly improves discrimination performance relative to single biomarker measurements. Using this approach, we observe promising discrimination of high-grade serous ovarian cancer versus benign ovarian masses and healthy women in a proof-of-concept clinical study.

Cancer remains a significant public health challenge globally and is the second leading cause of death in the United States after heart disease.^1^ Significant advances in the availability and efficacy of therapy have improved survival for some cancers, but the economic burden associated with cancer care has risen precipitously and is projected to approach $245 billion by 2030, with annual and cumulative costs of care increasing with cancer stage.^2^^,^^3^ Cancer diagnosed at an early stage is addressable through an array of treatment modalities and is typically more responsive to therapy.^1^^,^^4^ Given the significant clinical and economic benefits, the development and implementation of improved diagnostic tools for the early detection of cancer is a public health imperative.

**Key Points**

• Circulating extracellular vesicles (EVs) provide an abundant, biomarker-rich target for blood-based detection of small early-stage cancers given their molecular similarity to their cell of origin.
• Detection of multiple colocalized, membrane-associated proteins on single EVs significantly improves the signal to noise for detection of rare tumor-derived EVs in the large EV background found in blood.
• Proximity ligation quantitative PCR provides additional enhancement of both assay sensitivity and specificity.

To deliver net clinical benefit at a population scale, cancer screening tests must incorporate a foundational set of features. Tests intended for the early detection of cancer must have sufficient sensitivity to identify small, early-stage tumors, and specificity necessary to minimize overdiagnosis and false positives due to confounding comorbidities that may be present in the target population. Tests must be designed to facilitate broad adoption, including geographic and economic accessibility, and an experience that is convenient and noninvasive for the patient. Ideally, diagnostic tests also offer insights into the molecular mechanisms of the underlying cancer that can help guide clinical management. Historically, tests designated for screening in otherwise healthy asymptomatic individuals, whether deployed in an average- or high-risk patient population, have the added requirement of demonstrated mortality benefit, necessitating large, randomized control trials.^5^ To date, such trials have only been successfully completed for select high-prevalence cancers, resulting in routine screening recommendations only for colorectal, cervical, prostate, lung, and breast cancers. As a result, >70% of cancer deaths in the United States result from malignancies for which there is no recommendation for screening.^6^, ^7^, ^8^, ^9^, ^10^, ^11^

Traditional diagnostic modalities are not well suited for early cancer detection. Imaging-based techniques require specialized equipment that may not be broadly accessible, and often necessitate a separate clinical appointment. Low-cost imaging methods, such as low-dose computed tomography for lung cancer screening and trans-vaginal ultrasound for ovarian cancer screening, lack the specificity to accurately differentiate malignancies from benign masses.^12^, ^13^, ^14^ Although higher-resolution imaging techniques, such as positron emitted tomography/computed tomography, help to address this lack of specificity, the higher cost and more limited availability make them poorly suited as front-line cancer screening tools. Blood-based biomarker tests are an attractive alternative to imaging because of the low cost and broad accessibility, but single biomarker tests, including prostate-specific antigen for prostate cancer screening and cancer antigen 125 for ovarian cancer screening, have exhibited limited specificity.^6^^,^^7^^,^^15^^,^^16^ More recently, liquid biopsy approaches have been developed that are designed to measure genomic material released into circulation by apoptotic tumor cells. These methods leverage next-generation sequencing to measure a variety of genomic features, including the presence of somatic mutations, methylation patterns, and fragment lengths, in some cases integrating thousands of features into a classifier that yields a determination of the presence or absence of cancer.^17^^,^^18^ In addition to the cost and complexity associated with next-generation sequencing–based testing, these methods must overcome the biological limitation associated with the low abundance of circulating tumor DNA present in the context of small, early-stage tumors, and the limited stability of genomic material in standard blood collection tubes. These challenges may be addressed through the testing of large volumes of blood collected in specialized collection tubes designed to stabilize circulating DNA and RNA but introduce new logistic considerations.^19^, ^20^, ^21^, ^22^ Finally, classifiers trained on thousands of features run the risk of overfitting to chance effects, and even if proper statistical care is taken, the detected effects may be statistically valid but biologically false (eg, predicting slight differences in sample storage or handling instead of the disease, or other confounders).^23^, ^24^, ^25^ Although these challenges can be overcome with large sample sizes and rigorous validation protocols, it makes such classifiers difficult to develop, validate, and operationalize in practice.^26^, ^27^, ^28^

Extracellular vesicles (EVs) represent a diverse class of lipid-bilayer bounded nanoparticles ranging from 30 to 10,000 nm in diameter.^28^^,^^29^ They are released by all living cells and serve an array of biological functions, including cell-to-cell communication and the facilitation of cancer progression by establishing a metastatic niche and suppressing the immune response.^18^^,^^28^, ^29^, ^30^, ^31^, ^32^ EVs have been shown to contain numerous biomarkers derived from their cell of origin, including proteins, lipids, and nucleic acids.^32^ Tumor-associated EVs (tEVs) are a promising analyte for early cancer detection because of their abundance in blood and molecular similarity with their cell of origin. Unlike genomic-based detection methods, cell death is not required for the release of EVs.^21^^,^^33^ As a result, tEVs outnumber tumor-derived DNA copies in circulation by orders of magnitude in early-stage disease.^34^^,^^35^ This enables the utilization of smaller sample volumes (approximately 100 μL) relative to genomic-based liquid biopsy assays, which require several milliliters of blood per test.^36^ The high plasma EV concentration of approximately 10^10^ EVs per mL and estimated tEV shedding rates per cubic millimeter of tumor volume make EVs an abundant source of tumor-associated biomarkers to target in cancer screening assays designed for detection of smaller, early-stage tumors.^35^^,^^37^^,^^38^ Given their abundance, stability, and molecular similarity to their cell of origin, EVs offer a unique diagnostic test analyte that may offer advantages over other modalities.

Immuno-PCR and proximity ligation have been used for decades to improve the technical sensitivity and analyte specificity of immunoassays,^39^^,^^40^ and more recently these approaches have been used to evaluate EV-associated proteins as potential plasma biomarker targets for cancer detection.^41^^,^^42^

We hypothesized that detection of multiple cancer-associated biomarkers on the surface of individual tEVs could prove to be a powerful approach for detection of early-stage cancer. The novel assay method described herein interrogates the billions of EVs within a plasma or serum sample for the simultaneous detection of colocalized, cancer-associated biomarkers on the surface of individual tEVs. This immunoassay-based technique selectively captures EVs from specific cancer cells using antibody-functionalized magnetic beads targeting a cancer-associated surface biomarker and then detects them using oligo functionalized antibodies that recognize up to two additional cancer-associated surface biomarkers to generate a PCR-amplifiable product after proximity ligation. Following the rational design of biomarker combinations, the clinical feasibility of this approach was applied to the detection of high-grade serous ovarian cancer (HGSOC), the most prevalent and deadliest histotype of ovarian cancer.^43^ This method is an extensible platform technology that leverages colocalization for highly specific tEV detection and can in principle be applied to any solid tumor with available cancer-associated biomarkers.

## Materials and Methods

### Cell Culture

All human cancer cell lines used in this study, and their respective sources, are listed in Supplemental Table S1. SK-MEL-1 and SK-MES-1 were used under license from the Memorial Sloan Kettering Cancer Center (New York, NY). NCI-H146, NCI-H441, NCI-H520, and NCI-H1781 were developed by Dr. Adi Gazdar and Dr. John Minna and used under license from the National Cancer Institute (Bethesda, MD). COV362 and COV413A were used under license from the European Collection of Authenticated Cell Cultures (Porton Down, UK). OVSAHO and OVISE were obtained from Sekisui XenoTech (Kansas City, KS) under license from the Japanese Collection of Research Bioresources (Osaka, Japan). All cells were thawed, subcultured, and maintained in complete growth media, as recommended by the supplier. For EV production, cells were passaged into large-capacity tissue culture–treated flasks or dishes until 70% to 80% confluent. The flasks or dishes were then transitioned to EV-depleted fetal bovine serum (A2720801; Thermo Fisher Scientific, Waltham, MA) or serum-free Cancer Cell Line Media XF (CCLM XF; C-28077; PromoCell GmbH, Heidelberg, Germany), depending on the individual cell line, as follows. Growth medium was removed, and the cells were rinsed twice with one half volume of either complete medium supplemented with exosome-depleted (EV–) fetal bovine serum or CCLM XF serum-free media before being replaced in basal media with 10% EV– fetal bovine serum, or CCLM XF. After 48 hours, the conditioned medium (CM) was collected and cellular debris removed by centrifugation at 300 × *g* for 5 minutes, followed by two subsequent centrifugations at 1500 × *g* for 10 minutes each. All centrifugation steps were performed at 21°C. The cleared CM was transferred to 50-mL conical tubes in approximately 45-mL aliquots and stored at –80°C until needed for EV enrichment.

### Concentration of Conditioned Media

Typically, 200 mL of CM was thawed at room temperature and centrifuged at 1300 × *g* for 10 minutes to remove any insoluble debris. The supernatant was transferred via serological pipet to fresh 50-mL conical tubes and then recentrifuged at 2000 × *g* for 30 minutes. The supernatant was carefully transferred to a fresh tube and concentrated using Amicon Ultra Centrifugal filter units with a 10,000 molecular weight cutoff (Millipore-Sigma, St. Louis, MO). This process was repeated until all the retained material was concentrated to approximately 1 mL. All centrifugation steps were performed at 21°C unless specified otherwise.

### EV Isolation from Concentrated Conditioned Cell Culture Media or Plasma

EVs were enriched from 1 mL of concentrated CM, acid-citrate-dextrose human plasma, or potassium EDTA human plasma. In each instance, 1 mL of concentrated CM or plasma was applied to an Izon qEV original 70-nm Legacy size exclusion chromatography (SEC) column (ICO-70; Izon Science US, Medford, MA) following the vendor-recommended protocol. Briefly, qEV columns were allowed to equilibrate to room temperature and washed in 10 mL room temperature 1 × phosphate-buffered saline (PBS). Concentrated CM (described above) was applied to the top of the column, and the flow through material was discarded. Two milliliters of 1 × PBS was applied to the column and collected as the pre-EV fraction and analyzed as described below. EV-elution fractions (EV1, EV2…EV8) were then collected by applying 0.5 mL 1 × PBS sequentially until all desired fractions were collected. The pooled collection volume was determined on the basis of physical and functional characterization of each EV fraction. Concentrated CM EVs were quantified using the ZetaView Classic S model nanoparticle tracking analysis (NTA) instrument (Particle Metrix, Amersee, Germany), as described below before aliquoting and freezing at −807°C. The protocol for purifying plasma and CM EVs was identical, with the exception that 0.02 μmol/L filtered 1 × PBS, pH 7.4, was used for the concentrated CM EV purification (so particles in the PBS will not confound the downstream quantification) while unfiltered 1 × PBS, pH 7.4, was used to purify EVs from plasma.

### Western Blot Analysis of Human Cell Line or Plasma Extracellular Vesicle Fractions

Whole-cell extracts of COV413A cells were prepared by scrape harvesting 90% to 95% confluent cells in 1× Cell Lysis Buffer (number 9803; Cell Signaling Technology, Danvers, MA). Lysates were incubated at 4°C for 30 minutes before ultrasonic cavitation (3 × 15 seconds at 33% power) using an QSonica Q125 Ultrasonic Disruptor (QSonica, Newtown, CT) fitted with a 2-mm titanium probe tip. Protein concentrations were determined, and the lysate was further diluted to 1 mg/mL in 4× lithium dodecyl sulfate Sample Buffer (NP0008; Thermo Fisher Scientific). Human plasma lysate was prepared by mixing whole human plasma 1:1 with 4× lithium dodecyl sulfate Sample Buffer, followed by ultrasonic cavitation to homogenize the sample. Individual EV fractions from concentrated CM were diluted 1:1 in 4× lithium dodecyl sulfate Sample Buffer, and human plasma EVs were concentrated 10:1 before 1:1 dilution in 4× lithium dodecyl sulfate Sample Buffer. In all cases, samples were heated to 70°C for 5 minutes before use as recommended by the manufacturer. For PAGE and Western blot analysis experiments, 10 to 20 μL of whole cell extract, plasma lysate, or EV fraction was loaded alongside a biotinylated ladder and/or prestained protein molecular weight markers (catalog numbers 7727 and 59,329, respectively; Cell Signaling Technology) on NuPAGE Bis-Tris precast gels (Thermo Fisher Scientific). Gel percentage, running buffer, and transfer conditions were dictated by the molecular weight of the proteins being immunoblotted. In most cases, the authors used 1.0 mm, 4% to 12% Bis-Tris mini gels with MOPS-based running buffer (NP0001; ThermoFisher, Waltham, MA), according to the manufacturer's instructions. After PAGE, gels were either stained with Gel-Code Protein Stain (24,590; ThermoFisher) or transferred to 0.2-μm polyvinylidene difluoride membranes (1,620,174; Bio-Rad, Hercules, CA) using an XCell SureLock MiniCell (EI9051; ThermoFisher), according to the manufacturer's recommended procedure. After electrophoretic transfer, all membranes were blocked at room temperature for 1 hour in 1× Tris-buffered saline–Tween (TBST; number 9997; Cell Signaling Technology) supplemented with 5% nonfat dry milk (AB10109; American Bioanalytical, Canton, MA). Primary antibodies (Table 1) were incubated overnight (approximately 16 hours) in 1× TBST supplemented with either 5% nonfat dry milk or bovine serum albumin (numbers 9997 and 9998; Cell Signaling Technology) at 4°C with constant rocking, according to the manufacturer's recommendation. Each blot was washed thrice for 5 minutes in 1× TBST at room temperature before addition of horseradish peroxidase–conjugated secondary antibodies and horseradish peroxidase–anti-biotin antibody, both diluted in 1× TBST + 5% nonfat dry milk, for 1 hour at room temperature. Each blot was then washed thrice in 1× TBST for 5 minutes, and proteins were detected using either LumiGLO/Peroxide Reagent or SignalFire ECL reagent (7003 and 6883, respectively; Cell Signaling Technology) using an iBright imaging system (ThermoFisher). All images are presented with no adjustments (other than cropping to highlight the area of interest) and are typically between 5- and 15-minute exposures, depending on the signal strength.

**Table 1:** Antibodies Used for Western Blot Analysis in This Study

Antibody target	Antibody clone	Catalog no.	Vendor	Dilution	Antibody RRID
CD63	E1W3T	52090	CST	1:1000	AB_2924771
CD81	D3N2D	56039	CST	1:1000	AB_2924772
CD9	D801A	13174	CST	1:1000	AB_2798139
Flotillin	D2V7J	26836	CST	1:1000	AB_2773040
BST2	D5V5Z	19277	CST	1:1000	AB_2798815
FOLR1	EPR20277	ab235140	Abcam (Cambridge, UK)	1:1000	AB_2924727
TOM20	D8T4N	42406	CST	1:1000	AB_2687663
RPS3	D50G7	9538	CST	1:1000	AB_10622028
CD81	E2K9V	52892	CST	1:1000	AB_2924773
GAPDH	D16H11	5174	CST	1:1000	AB_10622025
MUC 1-C	D5K9I	16564	CST	1:1000	AB_2798765
Anti-rabbit IgG, HRP linked	Polyclonal	7074	CST	1:1000	AB_2099233
Anti-mouse IgG, HRP linked	Polyclonal	7076	CST	1:1000	AB_330924
Anti-biotin IgG, HRP linked	Polyclonal	7075	CST	1:1000	AB_10696897

BST2, bone marrow stromal antigen-2; CST, Cell Signaling Technology; FOLR1, folate receptor-α; GAPDH, glyceraldehyde-3-phosphate dehydrogenase; HRP, horseradish peroxidase; RPS3, ribosomal protein S3; RRID, Research Resource Identifier; TOM20, mitochondrial import receptor subunit TOM20 homolog.

### Characterization of EV Size Distribution and Concentration

Size distribution and particle counts were determined using a ZetaView NTA instrument (Particle Metrix, Ammersee, Germany) after enrichment of the EVs by SEC. ZetaView software 8.05.14 SP7 acquired and exported the data. The instrument was calibrated as recommended by the manufacturer before all measurements. Following SEC isolation and before NTA measurement, the isolated particles were diluted 1:250 to 1:2000, which allowed the instrument to accurately measure the particle distribution (50 to 200 particles per field of view). Manufacturer-recommended instrument settings were used for acquisition as follows: sensitivity (80), shutter (10 milliseconds/frame), minimum brightness (20), minimum area (100 pixels), maximum area (10,000 pixels), and frame rate (30). Eleven fields of view were analyzed per run, with outlier positions excluded automatically by the analysis software. The size distribution results were binned in 1-nm intervals from 1 to 1000 nm.

### Antibody Panels

All antibodies used for assay development were obtained from commercial sources (Table 2). Initially, approximately 50 different antibodies against the three biomarker targets [bone marrow stromal antigen-2/CD317/tetherin (BST2), folate receptor-α (FOLR1), and mucin-1 (MUC1)] were screened for functionality and compatibility using cell line EVs. Antibody clones that passed primary screening were further optimized to determine the optimal conjugation concentration of both probes and beads in biomarker-expressing cell lines versus biomarker-negative cell lines or buffer alone (no EV) controls.

**Table 2:** Antibodies Used for Capture and/or Detection in This Study

Name	Antibody clone	Vendor	Catalog no.	RRID
Recombinant anti-BST2/tetherin antibody	EPR20202-169	Abcam	ab243563	AB_2941823
Anti-BST2/tetherin antibody [EPR20202-150] - BSA and azide free	EPR20202-150	Abcam	ab243561	AB_2941851
Anti-human CD317	Y129 (HM1.24)	BD Biosciences (Franklin Lakes, NJ)	Custom order	Custom
BST2 antibody (4F6)	4F6	Novus Biologicals (Centennial, CO)	NBP2-29622	AB_2941840
BST2 monoclonal antibody (3H4)	3H4	Invitrogen (Waltham, MA)	H00000684-M02	AB_1136901
Purified anti-human CD317 (BST2, tetherin) antibody	RS38E	Bio Legend (San Diego, CA)	348,402	AB_10588013
CD317 (BST2, PDCA-1) monoclonal antibody (26F8), functional grade, eBioscience	26F8	Invitrogen Antibodies	16-3179-82	AB_1518775
Anti-BST2/tetherin antibody [EPR20202-169] - BSA and azide free	EPR20202-169	Abcam	ab243563	AB_2941823
Recombinant anti-BST2/tetherin antibody [EPR23597-202] - BSA and azide free	EPR23597-202	Abcam	ab272175	AB_2941830
Anti-BST2/tetherin antibody - BSA and azide free (capture)	EPR20202-150	Abcam	ab242967	AB_2941821
Anti-BST2/tetherin antibody - BSA and azide free (detector)	EPR20202-169	Abcam	ab243001	AB_2941822
Folate receptor α monoclonal antibody (548,908)	548,908	ThermoFisher	MA5-23917	AB_2609390
Anti-folate binding protein/FBP antibody [EPR23387-276], BSA and azide free	EPR23387-276	Abcam	ab273159	AB_2941825
Recombinant anti-folate binding protein/FBP antibody (detector) [EPR23387-239]	EPR23387-239	Abcam	ab278006	AB_2941829
Recombinant anti-folate binding protein/FBP antibody (capture) [EPR23387-232]	EPR23387-232	Abcam	ab278005	AB_2941828
Human FOLR1 antibody	548,908	R&D Systems (Minneapolis, MN)	MAB5646	AB_2278620
FOLR1 antibody (2 G5C12)	2 G5C12	Novus Biologicals	NBP2-61773	AB_2941842
Anti-folate binding Protein/FBP antibody [EPR20277] - BSA and azide free	EPR20277	Abcam	ab235140	AB_2924727
Anti-human folate receptor 1 recombinant antibody (farletuzumab)	Farletuzumab	Creative Biolabs (Shirley, NY)	TAB-113	AB_2941836
Monoclonal mouse anti human FOLR1/folate receptor α antibody	3 G12B7	LSBio (Shirley, MA)	LS-C682639	AB_2941839
Anti-folate binding protein/FBP antibody [LK26]	LK26	Abcam	ab3361	AB_303740
FOLR1 antibody	2B4B7	Proteintech	60307-1-Ig	AB_2881421
Human MUC-1 antibody	604,804	R&D Systems	MAB6298	AB_10640403
Anti-MUC1 antibody [EP1024Y] - low endotoxin, azide free	EP1024Y	Abcam	ab218998	AB_2941819
MUC1 (D9O8K) XP rabbit mAb	D9O8K	Cell Signaling Technology	14,161	AB_2798408
MUC1 (VU4H5) mouse mAb	VU4H5	Cell Signaling Technology	4538	AB_2148549
Mouse anti human CD227	VU-3C6	Bio-Rad (Hercules, CA)	6378-0135	AB_620067
MUC1 monoclonal antibody (GP1.4)	GP1.4	Invitrogen Antibodies	MA1-06503	AB_558126
MUC1 monoclonal antibody (GP1.4)	GP1.4	Novus Biologicals	NBP2-33174	AB_2941841
Epithelial membrane antigen (concentrate)	E29	Dako Cytomation (Santa Clara, CA)	M061301-2 (1 mL)	AB_2148557
Anti-human MUC1 recombinant antibody (1B2)	1B2	Creative BioLabs	TAB-439MZ	AB_2941838
Anti-MUC1 [12D10]	12D10	Absolute Antibody (Boston, MA)	Ab02843-1.1	AB_2941833
Human MUC-1 antibody	2336A	R&D Systems	MAB62981	AB_2941844
CD227 antibody | C595 (NCRC48)	C595 (NCRC48)	Bio-Rad	MCA1742	AB_322211
MUC1 antibody (SM3)	SM3	Novus Biologicals	NB120-22711	AB_789083
MUC1 monoclonal antibody (115D8)	115D8	Invitrogen	MA1-35039	AB_1070963
Anti-MUC1 antibody [115D8] - BSA and azide free	115D8	Antibodies.com LLC (St. Louis, MO)	A252591	AB_2941835
Anti-human MUC1 (CD227) antibody, clone 16A	16A	Stem Cell Technologies (Vancouver, BC, Canada)	60155	AB_2941845
Purified anti-human CD227 (MUC-1) antibody	16A	Bio Legend	355602	AB_2561642
Anti-human MUC1 therapeutic antibody (5E5)	5E5	Creative Biolabs	TAB-418MZ	AB_2941837
Anti-MUC1 antibody [HMFG1 (aka 1.10.F3)] - BSA and azide free	HMFG1 (aka 1.10.F3)	Abcam	ab230298	AB_2941820
Anti-MUC1 HMFG1 (1.10.F3)	HMFG1	Absolute Antibody	AB02206-1.1	AB_2941832
Anti-MUC1 antibody [HMFG2]	HMFG2	Abcam	ab245693	AB_2941824
Human MUC-1 antibody	604,804	R&D Systems	MAB6298	AB_10640403
CD81 monoclonal antibody (M38)	10630D	ThermoFisher	M38	AB_2532984
CD63 monoclonal antibody (Ts63)	10628D	ThermoFisher	Ts63	AB_2532983
Purified anti-human CD9 antibody	312,102	Bio Legend	HI9a	AB_314907

BSA, bovine serum albumin; BST2, bone marrow stromal antigen-2; FBP, folate binding protein; FOLR1, folate receptor-α; MUC1, mucin-1; RRID, Research Resource Identifier.

### Transmission Electron Microscopy of Cell-Line and Plasma EVs

Cancer cell-line and HGSOC cancer EVs were prepared by SEC, as described in EV Isolation from Concentrated Conditioned Cell Culture Media or Plasma, and sent to Alpha Nano Tech (Morrisville, NC) in 1× PBS, on dry ice, for transmission electron microscope imaging using a Gatan Orius SC1000 charge-coupled device camera with Gatan Microscopy Suite 3.0 software (Pleasanton, CA). Copper-carbon formvar grids were glow discharged immediately before loading with the sample. Samples were processed undiluted. Grids were floated on a 10-μL sample drop for 10 minutes, washed two times with water by floating on the drop of water for 30 seconds, and negatively stained with 2% uranyl acetate by floating on the drop of stain for 30 seconds. The grid was blot dried with Whatman paper and imaged with Jeol 1230 electron microscope. Pixel sizes for 400,000×, 800,000×, and 1,200,000× are 1.28, 0.7, and 0.47 nm, respectively.

### Superresolution Microcopy

EV biomarker colocalization was evaluated with direct stochastic optical reconstruction microscopy using an ONI Nanoimager S and an EV Profiler Kit [Oxford Nanoimaging Inc. (ONI), Oxford, UK]. For consistency, the top-performing capture and detection antibodies used in the PCR assay were also used for nanoimaging. Antibodies against BST2 and MUC1 were conjugated to Alexa Fluor 647 using the Zip Alexa Fluor Rapid Antibody Labeling Kit from Thermo Fisher Scientific (Z11235). FOLR1 was conjugated to Alexa Fluor 568 using the Alexa Fluor 568 Conjugation Kit (Fast) - Lightning-Link from Abcam (ab69821; Cambridge, UK). MUC1 was biotinylated for EV capture using Biotin Conjugation Kit (Fast, Type B) - Lightning-Link from Abcam (ab201796). EVs collected from conditioned media from COV413A or SK-MEL-1 cells were purified by SEC, as described above, characterized by NTA, then captured on imaging slides using Tetraspannin Trio (TetTrio) capture (EV Profiler 2 application kit; ONI) or MUC1 capture using the manufacturer-provided protocol. Briefly, the microfluidic imaging chip was functionalized with TetTrio biotinylated antibody cocktail or MUC1-biotin (2.5 μg/lane) to capture vesicles then incubated with 4 × 10^10^ COV413A or SK-MEL-1 derived EVs. After allowing the EVs to bind the surface, EVs were stained in ONI staining solution containing antibody cocktail consisting of MUC1-AF647 (5 μg/mL) or FOLR1-AF568 (2 μg/mL) and/or BST2-AF647 (5 μg/mL). All staining solutions also contained Pan EV-488 (PanEV; ONI) to identify membrane-containing particles. After washing, the chip was prepared according to the manufacturers' instructions and imaged. A total of 9000 frames at 40-millisecond exposure time were collected with 3000 frames each at 640, 561, and 488 nm at 38%, 30%, and 44% laser power, respectively. The CODI software package [*https://alto.codi.bio* (registration required), free beta version, release date June 2023] was used for subsequent image analysis using the settings indicated (Table 3). Pan EV-positive EVs that were triple positive for MUC1, BST2, and FOLR1 were defined as clusters identified by density-based clustering algorithm with a radius of ≤85 nm and with ≥5 localizations of BST2, Pan EV, and MUC1 and ≥10 localizations of FOLR1.

**Table 3:** AutoEV Image Analysis Settings

Attribute	Analysis settings
Drift correction	Performed automatically, no change necessary
Filtering	
Frame index	0-X
Photon count	200-5000
Sigma	75-200
*P* value	0.000001-0.05
Localization precision	0-20
DBSCAN clustering tool	
Distance	85 nm
Minimum samples	5
Minimum size	30
Cluster filtering	
Area	2500-150,000
Circularity	0.7-1
Radius of gyration	5-1,000,000
Density	0.0001-0.2
Counting tool	
PanEV (membrane stain)	5 to infinity
Tet-Trio (CD9 + CD63 + CD81)	10 to infinity
BST2	5 to infinity
FOLR1	10 to infinity
MUC1	5 to infinity
Image settings	Auto 1.5 Fixed 5-nm display sigma

BST2, bone marrow stromal antigen-2; DBSCAN, density-based spatial clustering of applications with noise; FOLR1, folate receptor-α; MUC1, mucin-1.

### Preparation of Antibody-Functionalized Magnetic Beads

Antibodies containing bovine serum albumin (BSA) were purified using protein A and G Spintrap purification kits (GE28-9031 to 34 or GE28-9031 to 32; Cytiva, Marlborough, MA), according to manufacturers' instructions. All antibodies used in the assay (Table 2) underwent a buffer exchange process to ensure that any carriers or impurities that might interfere with conjugation were removed. Up to 500 μg of antibody material was added to 2 mL of sterile 1× PBS (10,010,031; Thermo Fisher Scientific) in 30 KMW Amicon Ultra Filter Units (UFC203024; Sigma-Aldrich, St. Louis, MO) and centrifuged for 10 minutes at 4375 × *g*. This process was repeated six times, after which the antibodies were recovered and sterile filtered by centrifugation in 0.22 μmol/L Spin-X tube filters (8160; Corning-Costar, Corning, NY) for 2 minutes at 12,000 × *g*. Antibody concentrations were measured using the NanoDrop spectrophotometer (Thermo Fisher Scientific). Immunoaffinity magnetic beads were prepared by coating antibodies onto Dynabeads M-270 Epoxy beads using the Dynabeads Antibody Coupling Kit (14311D; Thermo Fisher Scientific), according to the manufacturer's instructions. Briefly, the beads were suspended at 10 mg/mL in C1 buffer and mixed thoroughly by vortexing. The 1-mL aliquots of the beads were transferred to 1.5-mL tubes, which were then placed on a DynaMag-2 magnetic rack (12321D; ThermoFisher) for 1 minute to separate the beads from the solution, and the supernatant was removed. C1 buffer and 20 to 160 μg of capture antibody, totaling a volume of 500 μL, were added and the beads were resuspended by gentle vortexing. After adding 500 μL C2 buffer, the beads were gently vortexed, and the tubes were placed on a HulaMixer (Thermo Fisher Scientific) for conjugation at 37°C for 19 ± 0.5 hours. The tubes were briefly centrifuged at 300 × *g* for 10 seconds, placed on the magnetic rack for 1 minute to separate the beads from the solution, and the supernatant was removed. The beads were washed using four cycles of resuspension and pulse centrifugation in Dynabeads wash solutions containing 0.05% Tween 20 (H5152; Promega, Madison, WI), followed by three washes in 1× PBS (10,010,072; Thermo Fisher Scientific) with 0.1% BSA (0332; VWR, Radnor, PA) and 0.1% Pluronic F-68 (P1169; Spectrum Chemical, New Brunswick, NJ). The antibody-functionalized magnetic beads were resuspended in 1 mL of 1× PBS, pH 7.4, with 50% glycerol (49,767; Sigma-Aldrich), 0.1% BSA, and 0.1% Pluronic F-68 and stored at –20°C.

### Preparation of DNA-Conjugated Antibodies

**Supplemental Figure S1:**
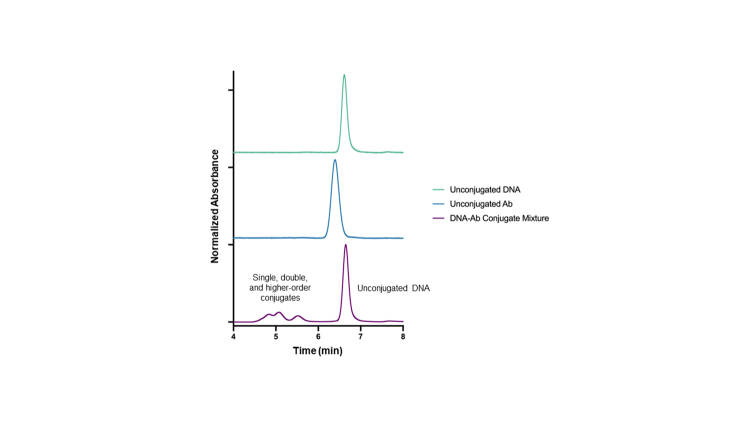
Characterization of DNA-antibody (Ab) conjugates. Size-exclusion chromatography traces (absorbance at 260 nm) for unconjugated DNA (**green line**; **top**), unconjugated antibody (**blue line**; **middle**), and a DNA-antibody conjugation mixture (**purple line**; **bottom**). DNA-Ab conjugates elute ahead of the unconjugated components in multiple peaks corresponding to different degrees of labeling. DNA is added in excess relative to unconjugated antibody, resulting in unconjugated DNA in the reaction mixture.

Antibodies were buffer exchanged and purified, if needed, as described in Preparation of Antibody-Functionalized Magnetic Beads. Antibodies (Table 2) were subsequently conjugated to DNA using a copper-free click chemistry reaction, as described previously.^44^ Oligonucleotides were synthesized and purchased as single-stranded DNA from Integrated DNA Technologies, Inc. (Coralville, IA) or Bio-Synthesis, Inc. (Lewisville, TX). Before performing the conjugation, the single-stranded DNA was annealed to form a double-stranded product. Complementary single-stranded DNA strands (Table 4) (strands 1 + 3 and strands 2 + 4) were mixed to a final concentration of 50 μmol/L in 1× PBS and annealed using a standard thermal cycler (Applied Biosystems, ProFlex PCR System, Carlsbad, CA). The annealing protocol was as follows: 95°C for 2 minutes, 90°C for 3 minutes, and then reducing the temperature by 5°C in 3-minute intervals until 25°C was reached. Monoclonal antibodies at 1 mg/mL in 1× PBS were incubated with DBCO-PEG5-NHS Ester linker (A102P; Click Chemistry Tools, Scottsdale, AZ) at a 4:1 mol/L ratio of cross-linker to antibody for at least 2 hours at room temperature. Following incubation, the antibody-linker solution was washed once using a 30-kDa Amicon Ultra-2 centrifugal filter (UFC203024; Sigma-Aldrich). Annealed DNA oligonucleotides with a 5′ azide modification (Integrated DNA Technologies) were added to the activated antibodies at a 3:1 mol/L ratio of oligonucleotide to antibody and were allowed to react overnight at room temperature. The conjugate concentrations were measured using the Qubit protein assay kit (Q33211; Thermo Fisher Scientific), according to the manufacturer's instructions, and the DNA-conjugated antibodies were stored at 4°C in 1× PBS. Select antibody-oligonucleotide conjugates were characterized by SEC using an Agilent 1260 Infinity II Bio-Inert system (Santa Clara, CA). Conjugates were analyzed using an AdvanceBio SEC 300 Å, 7.8 × 300 mm, 2.7 μm high-performance liquid chromatography column (PL1180-5301; Agilent Technologies) with PBS as the running buffer at a flow rate of 1 mL/minute. The chromatograms demonstrate a distribution of conjugate species (Supplemental Figure S1) containing single, double, and higher-order conjugates. The average degree of labeling was determined by removing unconjugated DNA by high-performance liquid chromatography, measuring the absorbance of the purified conjugates at 260 and 280 nm, and calculating the molar concentrations of DNA and antibody using previously measured extinction coefficients and the equations provided below.

(1) $$A_{260}=c_{mAb}\varepsilon_{mAb,260}l+c_{DNA}\varepsilon_{DNA,260}l$$

(2) $$A_{280}=c_{mAb}\varepsilon_{mAb,280}l+c_{DNA}\varepsilon_{DNA,280}l$$

**Table 4:** Oligonucleotide Sequences for Assay Probes, PCR Primers, and TaqMan Probes

Name	Sequence
Strand 1	5’-/Azide/CAGTCTGACACAGCAGTCGTGACTGGCTAGACAGAGGTGT-3′
Strand 2	5’-/Azide/GACCTGACCTACAGTGACCATTGGCTCCTGGTCTCACTAG-3′
Strand 3	5’-/Phos/GAGTACACCTCTGTCTAGCCAGTCACGACTGCTGTGTCAGACTG-3′
Strand 4	5’-/Phos/ACTCCTAGTGAGACCAGGAGCCAATGGTCACTGTAGGTCAGGTC-3′
Primer 1	5′-CAGTCTGACACAGCAGTCGT-3′
Primer 2	5′-GACCTGACCTACAGTGACCA-3′
TaqMan probe	5’-/56-FAM/TGGCTAGAC/ZEN/AGAGGTGTACTCCTAGTGAGA/31ABkFQ/-3′

Conjugation efficiency was similar among the antibodies characterized with average degree of labeling ranging between two and three DNA strands per antibody.

### Plasma Samples

The 24 healthy donor plasma samples were collected for Mercy BioAnalytics under a WCG Institutional Review Board–approved protocol (20212722). All individuals participating in the healthy collection study provided written informed consent, and the study complied with the Health Insurance Portability and Accountability Act and the Declaration of Helsinki. Each donor provided two tubes of blood, which were collected using a 10-mL collection volume K_2_EDTA tube (0265732; Thermo Fisher Scientific). After collection, each tube of K_2_EDTA plasma was inverted six to eight times, centrifuged (1500 × *g* at room temperature for 15 minutes) at the collection site within 60 minutes of collection to separate the plasma from the other blood components. The resultant plasma was divided into 1-mL aliquots in 2-mL cryovials, frozen, and stored at –80 °C. The frozen aliquots were shipped from the collection site to Mercy BioAnalytics on dry ice and stored at –80°C. A plasma pool from 90 healthy women was prepared in-house using samples purchased from ProteoGenex Inc. (Inglewood, CA). Late- and early-stage HGSOC cases were purchased from ProteoGenex, Inc. and Reprocell (Beltsville, MD). All ProteoGenex plasma samples from either K_2_EDTA or K_3_EDTA blood collection tubes were sourced from Moscow, Russia, in accordance with their ethics policy (ProteoGenex Inc.). Clinical annotation of the samples was also provided by ProteoGenex. Reprocell, Inc. samples were sourced from the United States in accordance with their bioethics policy (Reprocell) and collected in acid-citrate-dextrose blood collection tubes. Plasma samples from women with benign ovarian masses purchased from ProteoGenex were also sourced from Moscow, Russia, and collected in K_2_EDTA blood collection tubes. Patient sample attributes are shown in Supplemental Table S2.

### Antibody Selection

The top antibodies for the MUC1, BST2, and FOLR1 combination were then validated in human plasma and further down-selected based on those antibodies that produced the best discrimination of HGSOC from healthy women and those with benign adnexal masses.

### Assay Protocol for Measurement of Colocalized Biomarkers Using Immunocapture and Detection

**Figure 1:**
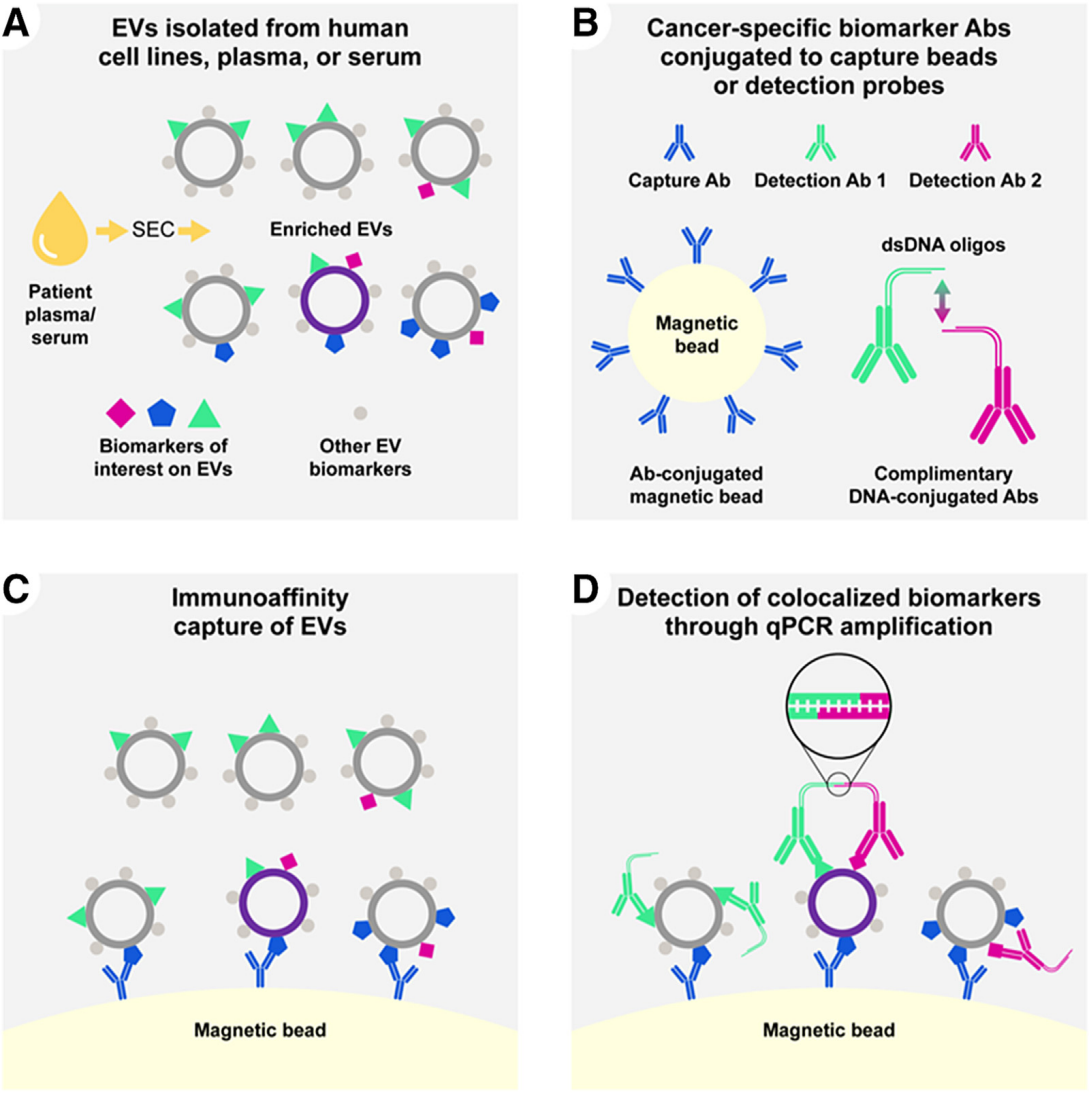
Overview of the assay method. Schematic representation of the assay workflow. **A:** Extracellular vesicles are enriched from human cell line conditioned media, plasma, or serum by size-exclusion chromatography (SEC). Enriched EVs contain cargo and surface biomarkers from their cell of origin. **B:** Antibodies targeting cancer-associated biomarker antibodies (Abs) are conjugated to magnetic beads (capture antibody) or double-stranded DNA (dsDNA) oligonucleotides (detection antibodies). **C:** SEC-enriched EVs are captured in solution with magnetic bead–antibody conjugates targeting a specific surface biomarker. **D:** Immunoaffinity captured EVs are incubated with detection antibodies conjugated to complementary double-stranded DNA probes. The dsDNA oligonucleotides contain single-stranded overhangs that ligate only when in proximity to a complementary probe to generate a template for PCR. The abundance of the detection biomarkers captured on the EVs is read out using quantitative PCR. Positive signal or low cycle threshold (C_T_) is the result of all three biomarker antibody epitopes being present simultaneously on the surface of the same EV, the absence of one or more biomarkers results in failed capture, or failed detection and low assay signal (high C_T_). qPCR, real-time quantitative PCR.

A conceptual schematic of the assay workflow is depicted in Figure 1. EVs from cultured human cell lines, human plasma, or serum are enriched using SEC (Figure 1A). For capture and detection of biomarkers on the surface of EVs, monoclonal antibodies specific to the extracellular domains of cancer-associated targets are conjugated to magnetic beads (capture antibody) or complementary dsDNA oligonucleotides (detection antibody), as described above and used as indicated in Figure 1B. Enriched EVs are isolated using magnetic bead–bound antibodies to the capture biomarker (Figure 1C), followed by detection with two antibodies to a second and third biomarker conjugated to complementary dsDNA oligonucleotides (Figure 1D). These complementary oligonucleotides hybridize and ligate if two DNA-conjugated antibodies are bound in proximity on the same EV (Figure 1D). The ligated DNA contains primer sites serving as a template for real-time quantitative PCR (qPCR) amplification and a fluorogenic probe binding site spanning the ligation junction to quantify the number of colocalization events in the sample. On the basis of this assay design, the complete set of biomarkers, detected by the antibody conjugated to the magnetic bead as well as the antibodies conjugated to the complementary oligonucleotides (a biomarker combination) must be present on the surface of a single EV to generate a signal.

Following EV isolation by SEC as described above, a concentrated buffer solution was added to the purified EVs to obtain working concentrations of 0.1% BSA, 0.1% Pluronic F-68, 100 μg/mL salmon sperm DNA (15632011; ThermoFisher), and 0.1% ProClin 950 (46878-U; Sigma-Aldrich). Antibody-functionalized magnetic beads directed against biomarker 1 were diluted 1:2 with 0.1% BSA, 0.1% Pluronic F-68, 100 μg/mL salmon sperm DNA, and 0.1% ProClin 950, and 4.8 μL aliquots of the diluted beads per well (corresponding to 16 μg of beads/well) were added to the wells of a 96 deep-well KingFisher plate. A total of 250 μL of the diluted, purified EVs was added to each well of a deep-well KingFisher plate. The sample plate was covered with an adhesive plastic film, was placed on an Eppendorf ThermoMixer C (Eppendorf, Enfield, CT), and incubated at 21°C for 2 hours, mixing at 1100 × *g*. During this incubation, all the wash and reaction plates required for the assay were prepared and are described in Table 5.

**Table 5:** Configuration and Composition of the 96-Well KingFisher Plates

Reaction plate type	No.	Volume/well	Components
Antibody plate	1	100 μL	DNA-conjugated antibody, 0.1% BSA, 0.1% Pluronic F-68, 100 μg mL^−1^ salmon sperm DNA
TEPBS wash	2	1 mL	1× PBS, pH 7.4, 0.05% Tween 20, 5 mmol/L EDTA
PBST wash	1	1 mL	1× PBS, pH 7.4, 0.05% Tween 20
Ligation plate	1	100 μL	75,000 units mL^−1^ of T7 DNA ligase (M0318; New England Biolabs, Ipswich, MA), 33 mmol/L Tris-HCl, 5 mmol/L MgCl_2_, 0.5 mmol/L ATP, 0.5 mmol/L DTT, 3.75% PEG 6000, 0.5% Tween 20
PBST transfer plate	1	150 μL	1× PBS, pH 7.4, 0.05% Tween 20
qPCR plate	1	30 μL	TaqMan Fast Advanced Master Mix (4444557; Applied Biosystems, Carlsbad, CA), 500 nmol/L primers, 200 nmol/L TaqMan probe

BSA, bovine serum albumin; DTT, dithiothreitol; PBS, phosphate-buffered saline; PBST, PBS-Tween; qPCR, real-time quantitative PCR; TEPBS, Tween-EDTA-PBS.

After the 2-hour EV-bead–binding incubation was complete, a KingFisher Flex System (Thermo Fisher Scientific) was loaded with the sample plate, the antibody-probe plate, the wash plates, the ligation reaction plate, and the PBS-Tween transfer plate, and the custom KingFisher program initiated. Briefly, the beads are magnetically captured and incubated in the antibody-probe plate for 32 minutes 50 seconds, then washed twice in Tween-EDTA-PBS for 3 minutes 30 seconds, then washed in PBS-Tween for 3 minutes 30 seconds and placed in the ligase reaction plate for 21 minutes 40 seconds. Finally, the beads are transferred into a shallow plate containing PBS-Tween. The beads are then manually transferred to a qPCR plate using a KingFisher Flex 96 PCR magnetic head. qPCR plates were covered with a clear plastic adhesive and placed into QuantStudio Dx Real-Time PCR instruments (Thermo Fisher Scientific) run using the following amplification conditions: 50°C for 2 minutes, 95°C for 2 minutes, 40 cycles at 95°C for 5 seconds, and 60°C for 20 seconds. See Table 4 for primer and TaqMan probe sequences. All samples were run in either duplicate or triplicate, and those showing a cycle threshold (C_T_) spread >3 among the replicates were excluded from subsequent data analysis.

### Detergent Disruption of EVs

To demonstrate the assay is EV based, a detergent-sensitivity experiment was performed. Exposure of EVs to detergent should disrupt their membranes, resulting in loss of assay signal. As described in the paragraph above, EVs purified with SEC were diluted in a buffer containing 0.1% BSA, 0.1% Pluronic F-68, 100 μg/mL salmon sperm DNA, and 0.1% ProClin 950. Antibody-functionalized beads were used to capture the purified EVs in a 2-hour incubation under agitation. The beads with the captured EVs were then transferred, using the KingFisher Flex system, to deep-well plates containing 0.1% BSA, 0.1% Pluronic F-68, 100 μg/mL salmon sperm DNA, 0.1% ProClin 950, and 0.075% Triton X-100 (T8787; Sigma-Aldrich). The plates were incubated for 30 minutes, at 21°C, under agitation (1100 × *g*) in a ThermoMixer C instrument to allow for EV lysis. After incubation with detergent, the deep-well plate containing the beads was washed and read out with detection antibodies to either tetraspanins or the indicated biomarkers using the assay protocol described above.

### Statistical Analysis

Data are presented as PCR C_T_. When assay data were collapsed to a single value, the median of replicates was displayed. Cell line gene expression and mass spectrometry data were obtained from the Cancer Cell Line Encyclopedia. Mass spectrometry data were not available for all cell lines. Heat map data are ordered by signal strength. Heat maps containing multiple data types were row scaled with a cap of ±3 SDs to enable comparisons between different data modalities. When only gene expression was shown, heat maps were ordered to match the cell lines displayed in the figure, log2 transcripts per million (TPM) displayed, and no scaling was used. Correlation analysis between assay signal and gene expression or mass spectrometry data was done using Spearman rank correlation. Assay sensitivity at 96% specificity was calculated using the epiR R package version 2.0.40, and receiver operating characteristic (ROC) curves were generated with the R package ROCit version 2.1.1. All analyses were performed in R version 4.0.5.

## Results

### Identification and Selection of Biomarkers

**Figure 2:**
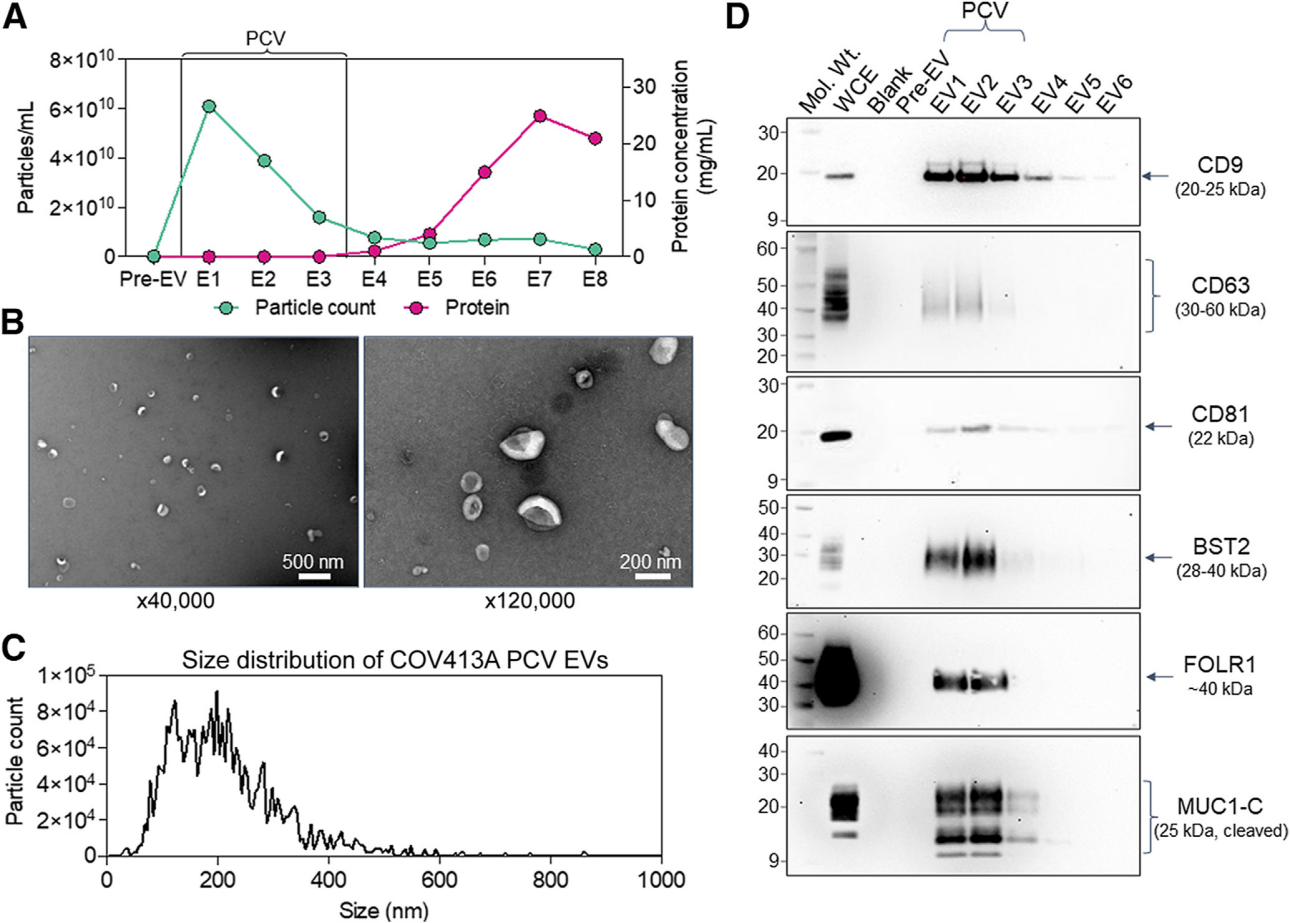
Characterization of human cancer cell line extracellular vesicles. Size-exclusion chromatography (SEC)–enriched EV fractions from human ovarian cancer cell line (COV413A) conditioned media were characterized according to minimal information for studies of extracellular vesicles guidelines. **A:** Nanoparticle tracking analysis (particle count; **greenline**) and protein quantification (protein; **pinkline**) of each fraction collected from the SEC column demonstrating the bulk of the nanoparticles elute in EV1 to EV3, distinct from the bulk of soluble protein (EV6 to EV8). The first fraction (Pre-EV) is the sum of the void and wash volumes of the column, which generally contains larger particles, such as lipoproteins, which are not observed in cell line conditioned media. The purified collection volume (PCV), fractions EV1 to EV3, containing the EVs used in downstream assay steps are highlighted. **B:** Transmission electron microscopy of EVs derived from the total PCV (pooled fractions EV1 to EV3) from COV413A conditioned media at two different magnifications demonstrates intact EVs ranging from approximately 40 to 300 nm. **C:** Particle size distribution of pooled SEC fractions EV1 to EV3 demonstrating particles consistent with the size range of small EVs ranging from approximately 40 to 400 nm. **D:** Western blot analysis of each of the initial fractions from the SEC column (Pre-EV, EV1 to EV6) from COV413A conditioned media demonstrating the presence of known EV surface biomarkers (CD9, CD63, and CD81), as well as bone marrow stromal antigen-2 (BST2), folate receptor-α (FOLR1), and mucin-1 (MUC1). Note that an antibody detecting the cleaved fragment of MUC1 (MUC1-C) was used to facilitate immunoblotting because of the high molecular weight of MUC1 (250 to 500 kDa). Whole cell extracts (WCEs; 10 μg total protein per lane) from COV413A were analyzed in parallel as a control for protein expression and biotinylated molecular weight (Mol. Wt.) markers were used to determine apparent molecular mass. **D:** Source data: uncropped images from the Western blot analyses shown in Figure 2. Immunoblot analysis of each of the initial fractions from COV413A cell line conditioned media concentrated and purified by SEC, as described in *Materials and Methods*. Size exclusion fractions (Pre-EV, EV1 to EV6) were evaluated for the presence of known EV surface biomarkers CD9, CD81, CD63, as well as BST2, FOLR1, and MUC1. Note that an antibody detecting MUC1-C was used to facilitate immunoblotting because of the high molecular weight of MUC1 (250 to 500 kDa). WCEs; (10 μg total protein per lane) from COV413A were analyzed in parallel as a control for protein expression, and biotinylated Mol. Wt. markers were used to indicate the apparent molecular mass. Scale bars: 500 nm (**B**, **left panel**); 200 nm (**B**, **right panel**).

**Supplemental Figure S2:**
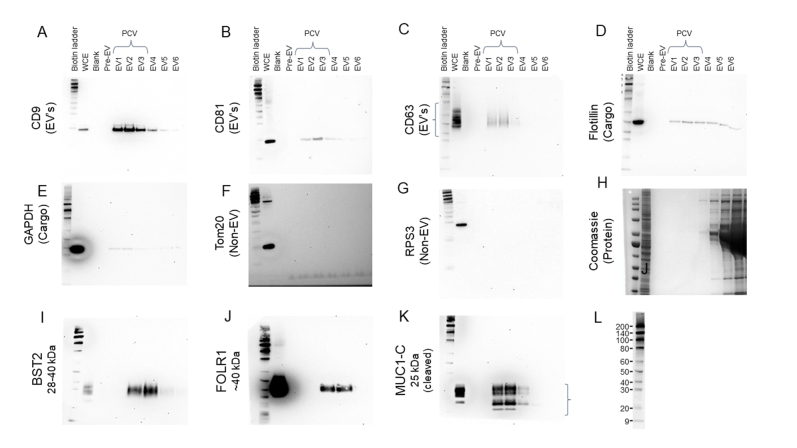
Expression of EV and ovarian cancer biomarkers in EV fractions. **A**–**G**: Full-panel blots of data shown in Figure 2 and additional Western blot analysis of whole cell extract (WCE) from COV413A cells and size-exclusion chromatography (SEC) purified fractions from COV413A conditioned media (EV1 to EV6) using antibodies against EV surface biomarkers CD9 (**A**), CD81 (**B**), and CD63 (**C**); EV cargo biomarkers flotillin (**D**) and glyceraldehyde-3-phosphate dehydrogenase (GAPDH; **E**) demonstrate the EVs were intact during enrichment; and non-EV internal membrane proteins mitochondrial import receptor subunit TOM20 homolog (TOM20) (**F**) and ribosomal protein S3 (RPS3) (**G**) demonstrate the EV fractions are the result of EV biogenesis and do not include intracellular vesicles. **H:** Total protein staining (Coomassie) was used to validate SEC separation of the EV fractions from the soluble protein component and was confirmed by measuring the protein concentration in each fraction (Figure 2). **I**–**K:** Full-panel blots of data shown in Figure 2 demonstrating abundance of bone marrow stromal antigen-2 (BST2; **I**), folate receptor-α (FOLR1; **J**), and mucin-1 (MUC1)-C (**K**) in COV413A EVs. **L:** Representative molecular weight markers with indicated observed molecular weights are shown as a reference. Source data: uncropped blot images. **A**–**G**: Western blot analysis of WCE from COV413A cells and SEC purified fractions from COV413A conditioned media (EV1 to EV6) using antibodies against EV surface biomarkers CD9 (**A**), CD81 (**B**), and CD63 (**C**); EV cargo biomarkers flotillin (**D**) and GAPDH (**E**) to demonstrate the EVs were intact EVs during enrichment; and non-EV internal membrane proteins Tom20 (**F**) and RPS3 (**G**) to demonstrate the EV fractions are the result of EV biogenesis and do not include intracellular vesicles. **H:** Total protein staining (Coomassie stain) was used to validate SEC separation of the EV fractions from the soluble protein component and was confirmed by measuring the protein concentration in each fraction as shown in Figure 2. **I**–**K:** Full-panel blots of data shown in Figure 2 demonstrating abundance of BST2 (**I**), FOLR1 (**J**), and MUC1-C (**K**) in COV413A EVs. Biotinylated molecular weight markers were detected using anti-biotin horseradish peroxidase antibody and are indicated on each panel.

For these studies, the authors focused on three biomarkers, BST2, FOLR1, and MUC1, all of which are cell surface proteins previously shown to be overexpressed in ovarian cancer.^45^, ^46^, ^47^, ^48^, ^49^, ^50^, ^51^, ^52^, ^53^ BST2 is a type II transmembrane protein shown to promote metastasis of human cancer cell lines and is highly expressed on a variety of human cancer types, including ovarian.^45^^,^^54^^,^^55^ FOLR1 is a glycosylphosphatidylinositol-anchored surface protein and a well-established diagnostic and therapeutic target for HGSOC among other cancers.^47^^,^^56^^,^^57^ MUC1 is a large, heavily glycosylated transmembrane protein cleaved at the plasma membrane to produce a noncovalent heterodimer of α and β (MUC1-C) subunits and is a well-established biomarker for the detection of ovarian neoplasms.^50^^,^^58^^,^^59^ Protein expression of BST2, FOLR1, and MUC1 was demonstrated by Western blot analysis to be present in the EV fractions from human ovarian cancer cell lines (Figure 2) and human plasma (data not shown) enriched by size exclusion. Evaluation of additional minimal information for studies of extracellular vesicles protein markers,^60^ the presence of EV cargo proteins (flotillin, glyceraldehyde-3-phosphate dehydrogenase), and absence of non-EV biomarkers [ribosomal protein S3 (RPS3), mitochondrial import receptor subunit TOM20 homolog (TOM20)] can be found in Supplemental Figure S2.

### Analyte Characterization

**Supplemental Figure S3:**
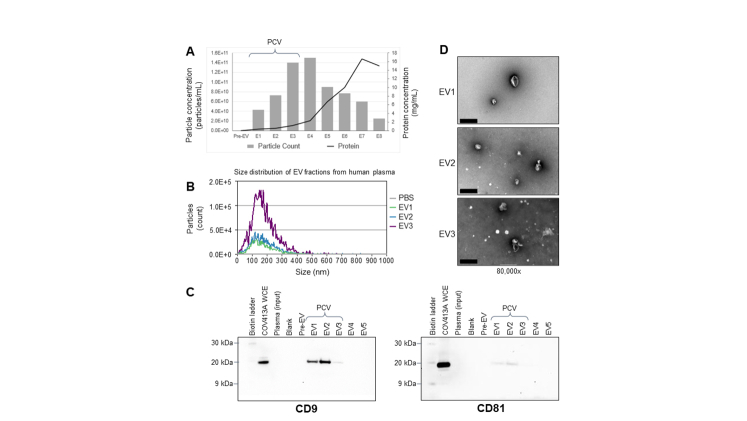
Characterization of human plasma EVs. EVs were enriched from a pool of healthy human plasma samples by size-exclusion chromatography (SEC). Individual fractions were collected and subject to nanoparticle tracking analysis (NTA). **A** and **B:** Unconcentrated SEC fractions (EV1, EV2, and EV3) were characterized to determine particle counts (**A**) and overall size distribution (**B**) of the enriched nanoparticles. **A:** Plot showing nanoparticle counts (gray bars) and protein concentration (**black line**) for the EV-containing SEC fractions from human plasma. The pooled collection volume (PCV), consisting of fractions EV1 to EV3, used in the downstream assay, are highlighted. The Pre-EV sample is a sum of the void volume and the wash fraction containing lipoproteins and larger, non-EV particles (data not shown). **B:** NTA size-distribution plot of individual fractions EV1, EV2, and EV3 demonstrating particles within the size range of 40 to 400 nm. Buffer alone [phosphate-buffered saline (PBS)] was used to determine the background. **C:** In parallel, concentrated SEC fractions were analyzed by Western blot analysis against normal human plasma (input) and COV413A whole cell extract (WCE) as a positive control. CD9 (**left panel**) and CD81 (**right panel**) are undetectable in the input sample because of the low relative abundance in human plasma. The PCV is indicated demonstrating CD9 and CD81 presence in fractions EV1 to EV3. **D:** Representative transmission electron microscopy images of EVs present in each of the three PCV fractions (EV1, EV2, and EV3) are shown, indicating intact particles between 40 and 400 nm. **C:** Source data: uncropped images from Western blot analyses. Concentrated SEC fractions from human plasma were analyzed by Western blot analysis against normal human plasma (input) and COV413A WCE as a positive control. Uncropped chemiluminescent images of immunoblots using CD9 (**left panel**) and CD81 (**right panel**) are shown. Note that both proteins are undetectable in the input sample because of the low relative abundance in human plasma but are enriched in the EV-bearing fractions EV1 to EV3. Scale bars = 500 nm (**D**). Original magnification, ×80,000 (**D**).

EVs enriched by SEC from human cell line conditioned media (Figure 2A and Supplemental Figure S2) and human plasma (Supplemental Figure S3) were characterized according to minimal information for studies of extracellular vesicles 2018 guidelines^60^ by NTA, transmission electron microscopy, and Western blot analysis to evaluate particle size, number, morphology, and phenotype. NTA for EVs from human cell lines (Figure 2, A and C) and human plasma (Supplemental Figure S3, A and B) demonstrates EV SEC fractions 1 to 3 (EV1-3) contain particles ranging in size from 40 to 400 nm, consistent with the known size distribution for EVs. The authors selected and pooled EV1-3 for immunocapture and detection in their assay. In human plasma, EV4 was excluded because of the higher background introduced by the abundance of soluble proteins in human plasma (Supplemental Figure S3A) and absence of EV biomarkers, CD9 antigen (tetraspanin-29/motility-related protein 1 (MRP-1)/CD9) and CD81 antigen (tetraspanin-28/target of antiproliferative antibody 1 (TAPA1)/CD81), as determined by Western blot analysis (Supplemental Figure S3C). The fundamental differences in NTA counts demonstrated between human plasma (Supplemental Figure S3) and human cell line conditioned media (Figure 2) is explained by the difference in heterogeneity between nanoparticles derived from human blood versus those enriched from a clonal cell line in culture.^61^^,^^62^ Enriched nanoparticles from both cell line and plasma EV fractions were further characterized by transmission electron microscopy, demonstrating lipid-bounded nanoparticles ranging from 40 to 400 nm, consistent with the NTA results (Figure 2B and Supplemental Figure S3D).^62^^,^^63^ Additionally, Western blot analysis was used to confirm the presence of well-established EV markers and absence of non-EV proteins as well as the presence of BST2, FOLR1, and MUC1 proteins within the EV fraction (Figure 2D and Supplemental Figure S2).

### Antibody Screening

**Supplemental Figure S4:**
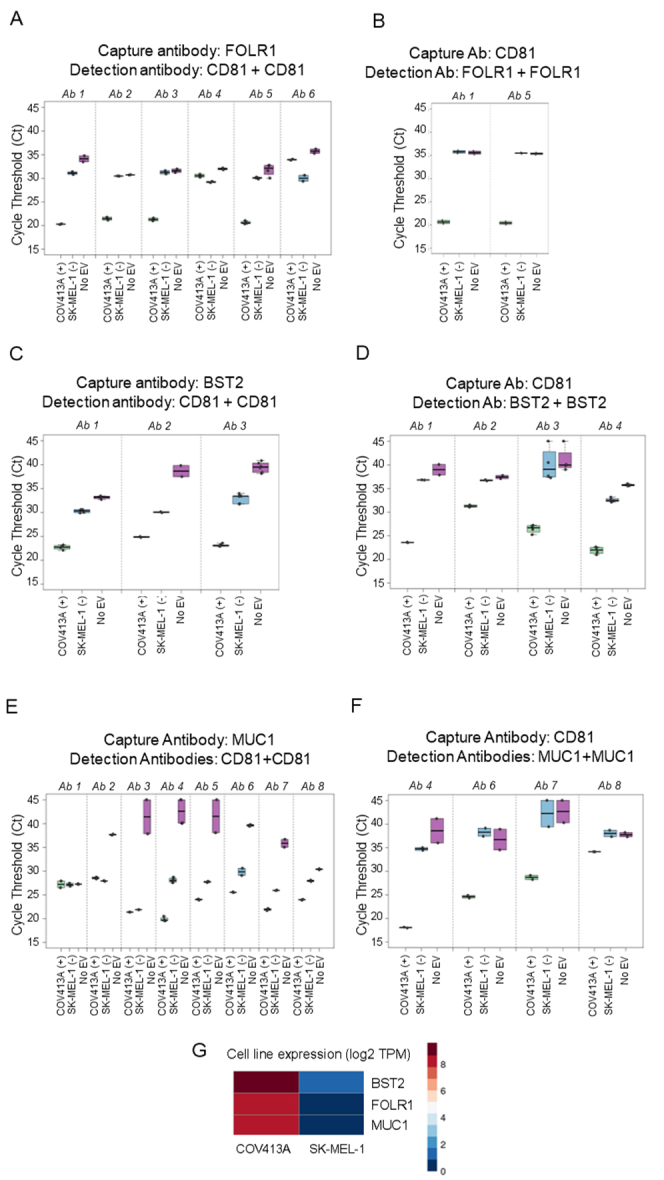
Screening antibodies (Abs) for functionality and specificity for capture and detection of EVs. **A**–**F:** Distinct monoclonal antibody clones against folate receptor-α (FOLR1; **A** and **B**), bone marrow stromal antigen-2 (BST2; **C** and **D**), and mucin-1 (MUC1; **E** and **F**) were screened for their ability to capture (**A**, **C**, and **E**) and detect (**B**, **D**, and **F**) size exclusion chromatography–purified EVs from human cell lines known to be positive [COV413A (+)] or negative [SKMEL1 (-)] for FOLR1, BST2, and MUC1. **A**, **C**, and **E:** Each antibody was independently conjugated to magnetic beads at a fixed density and used to capture cell line EVs in solution. Captured EVs were detected using complementary anti-CD81 double-stranded DNA-conjugated detection antibodies. Antibody clones demonstrating both functionality (greatest signal to noise between the positive and negative cell lin**e**) and specificity (low C_T_ in positive cell line and high C_T_ in negative cell line) were further tested and optimized (data not shown). No EV controls were used to eliminate cross-reactive or sticky antibodies. On the basis of performance in immunocapture and other criteria, selected antibodies were subsequently tested for their ability to function as a probe to detect cell line EVs captured using anti-CD81 conjugated beads. **B**, **D**, and **F:** EVs from COV413A and SKMEL were captured with anti-CD81 bead conjugates and read out in the assay using monoclonal anti-FOLR1 (**B**), anti-BST2 (**D**), or anti-MUC1 (**F**) detection antibodies. Antibodies demonstrating a high degree of functionality and specificity were subject to additional testing and optimization (data not shown). **G:** mRNA expression data (Cancer Cell Line Encyclopedia) for each biomarker are shown in the heat map, illustrating negative and positive expression of BST2, FOLR1, and MUC1 in SK-MEL-1 and COV413A, respectively.

Commercially available monoclonal antibodies to FOLR1, BST2, and MUC1 were screened for their ability to capture or detect EVs enriched from human cell lines predicted to express high (positive cell line) or low/no (negative cell line) levels of the selected biomarker (Supplemental Figure S4). Representative screening data for anti-FOLR1 antibodies used for capture (Supplemental Figure S4A) and detection (Supplemental Figure S4B) are shown using EVs derived from COV413A (FOLR1-positive) and SK-MEL-1 (FOLR1-negative) cell lines. Of the six antibodies screened for capture using CD81 antibodies for detection, antibodies 1 and 5 were selected for additional characterization based on the signal to background difference between the positive and negative cell line among other criteria. Antibodies 4 and 6 illustrate examples of reagents that failed validation in the capture assay because of lack of signal in the positive cell line (Supplemental Figure S4A). Antibodies 1 and 5 were further validated for detection using anti-CD81 conjugated beads for capture before detection with anti-FOLR1 antibodies (Supplemental Figure S4B). As with capture performance, both antibodies perform well in the detection step, as illustrated by their ability to distinguish between FOLR1-positive (COV413A) and FOLR1-negative cell line EVs (SK-MEL-1). Screening data for antibodies against BST2 and MUC1 described in this study are shown in Supplemental Figure S4, C–F. Note that despite moderate FOLR1 mRNA expression in NCI-H1819 cells, proteomics and Western blot analysis data both suggest that this cell line may not express FOLR1 protein. SK-MEL-1, which expresses low levels of each marker, was used as a negative control. Buffer alone (no EV controls) was used to eliminate antibodies incompatible with the assay because of non-specific binding to the beads or other antibodies under the assay conditions.

### Cancer Biomarkers Are Colocalized on the Surface of EVs

**Figure 3:**
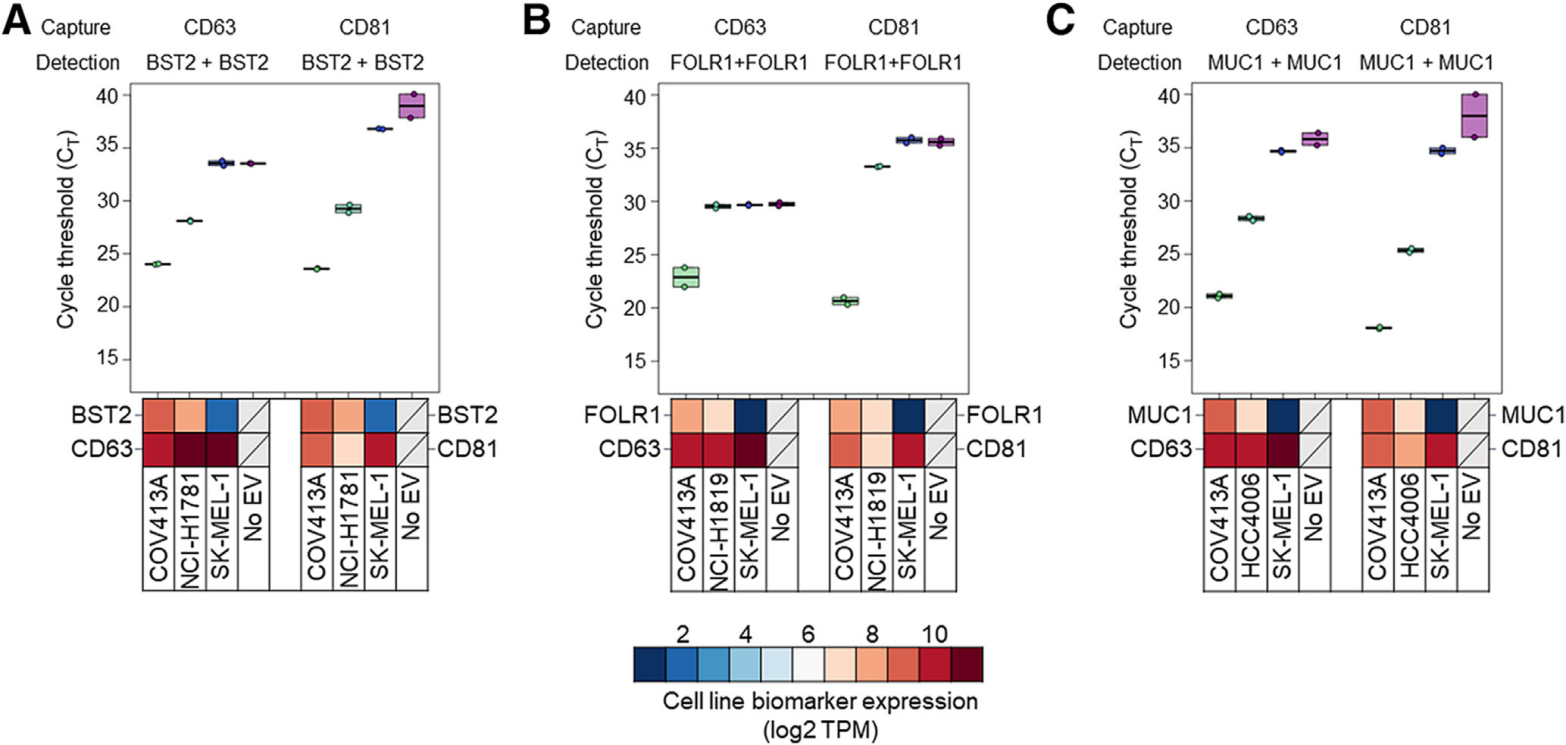
Bone marrow stromal antigen-2 (BST2), folate receptor-α (FOLR1), and mucin-1 (MUC1) are colocalized with CD63 and CD81 in cell line EVs. EVs derived from cell lines predicted to express high (red), medium (white), or low (blue) levels of the indicated biomarkers based on mRNA analysis were captured with antibody-bead conjugates using anti-CD63 or anti-CD81 antibodies. Detection of bound EVs was determined using oligo-conjugated antibodies against BST2 (**A**), FOLR1 (**B**), or MUC1 (**C**). COV413A EVs were used as a positive control because this line expresses high levels of all three biomarkers studied. For BST2, FOLR1, and MUC1, a second positive cell line, NCI-H1781, NCI-H1819, and HCC-4006, respectively, was also included as a control. Heat maps were included to demonstrate relative expression levels [log2 transcripts per million (TPM)] for each marker, and expression data are derived from the Cancer Cell Line Encyclopedia database at the Broad Institute (*https://sites.broadinstitute.org/ccle/datasets*, last accessed March 3, 2024).

To demonstrate colocalization of cancer-associated biomarkers with canonical EV biomarkers, the assay was applied to EVs derived from human cell lines known to express high or low levels of each cancer-associated biomarker. After capturing SEC-enriched EVs with antibodies directed against canonical EV tetraspanin biomarkers (CD63 antigen/tetraspanin-30/lysosomal-associated membrane protein 4/lysosomal integral membrane protein 1/CD63, or CD81), bound EVs were detected using antibodies directed against the extracellular domains of BST2, FOLR1, or MUC1 (Figure 3). Only EVs derived from cell lines positive for both the tetraspanin and the cancer-associated biomarker resulted in a strong qPCR signal.

**Figure 4:**
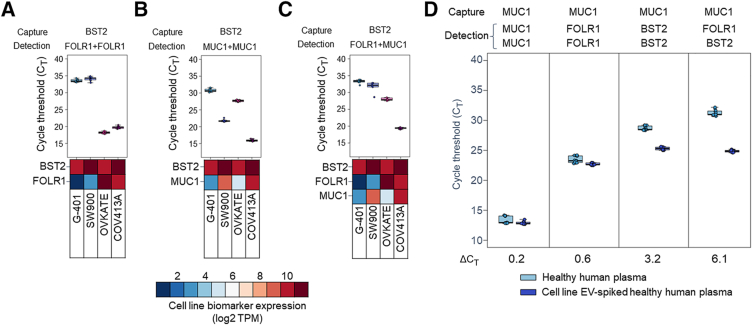
Colocalization of biomarkers is required for assay performance and improves signal/noise ratio. All three biomarkers must be present on the same EV for productive readout. If only two biomarkers are present on the EVs (one capture + one detect), no ligation occurs, and C_T_ is high. **A**–**C:** Doublet [ie, bone marrow stromal antigen-2 (BST2) capture, folate receptor-α (FOLR1) + FOLR1 detection or BST2 capture, mucin-1 (MUC1) + MUC1 detection; **A** and **B**] or triplet combinations (BST2 capture, FOLR1 + MUC1 detection; **C**) of antibodies tested on cell line EVs from COV413A (BST2^high^/FOLR1^high^/MUC1^high^), OVKATE (BST2^high^/FOLR1^high^/MUC1^low^), SW900 (BST2^high^/FOLR1^low^/MUC1^high^), and G-401 (BST2^high^/FOLR1^low^/MUC1^low^) showing that doublets of BST2 capture/FOLR1 + FOLR1 detection (**A**), BST2 capture/MUC1 + MUC1 detection (**B**), or a triplet of BST2 capture/FOLR1 + MUC1 detection (**C**) only yield positive qPCR signal in cell lines with matching high biomarker expression. Biomarker mRNA abundance (log2 TPM) is represented for each biomarker in each cell line. **D:** Signal from cell line EV-spiked (dark blue) or unspiked (light blue) healthy human plasma from a representative set of one-biomarker (MUC1 capture, MUC1 + MUC1 detection), two-biomarker (MUC1 capture/FOLR1 + FOLR1 detection or MUC1 capture/BST2 + BST2 detection), and three-biomarker (MUC1 capture/FOLR1 + BST2 detection) combinations. Increasing the number of unique, colocalized biomarkers results in reduced healthy background and improved discrimination of positive cancer cell line EV-spiked plasma. Signal difference (ΔC_T_) between unspiked healthy human plasma and spiked plasma is indicated below each plot.

Assay signal operates akin to a Boolean logic AND gate, whereby all three surface epitopes must be confined to the same EV for PCR template generation to occur. To demonstrate this, the authors characterized EVs derived from four human cancer cell lines (COV413A, OVKATE, SW900, and G-401) exhibiting a range of BST2, FOLR1, and MUC1 expression levels (Figure 4, A–C). The authors tested the functionality of the assay using two-biomarker (BST2 capture/FOLR1 + FOLR1 detection and BST2 capture/MUC1 + MUC1 detection) and three-biomarker (BST2 capture/FOLR1 + MUC1 detection) combinations in EVs from cell lines that were BST2^high^/FOLR1^high^/MUC1^low^ (OVKATE), BST2^high^/FOLR1^low^/MUC1^high^ (G-401 and SW900), and BST2^high^/FOLR1^high^/MUC1^high^ (COV413A) to demonstrate that assay signal is dependent on all of the capture and detection biomarkers being present for signal. Cell line EVs were tested at an equivalent concentration of 1.6 × 10^9^ particles/mL. Signal was observed to correlate with biomarker colocalization in the expected patterns (Figure 4). BST2 capture/FOLR1 + FOLR1 detection and BST2 capture/MUC1 + MUC1 detection generated strong signals for the cell lines that express both biomarkers (COV413A/OVKATE and COV413A/SW900, respectively). Weak BST2 capture/FOLR1 + FOLR1 detection and BST2 capture/MUC1 + MUC1 detection signals were observed for cell lines expressing low levels of at least one marker (SW900/G-401 and OVKATE/G-401, respectively). The three-biomarker combination BST2/FOLR1/MUC1 showed a clear separation between COV413A EVs (triple positive) and cell lines missing at least one biomarker, suggesting assay signal is only robust when all biomarkers are colocalized on the same EV.

### Detecting Multiple Colocalized Biomarkers on EVs Improves the Signal/Noise Ratio

**Supplemental Figure S5:**
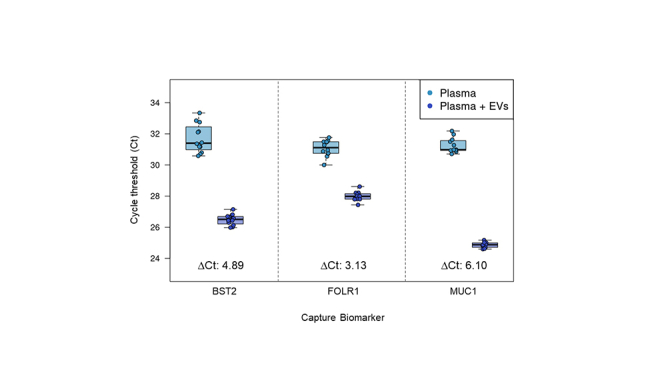
Impact of assay configuration on discrimination of spiked and unspiked plasma. Assay signal from COV413A cell line EV-spiked and unspiked human plasma for the biomarker combination bone marrow stromal antigen-2 (BST2)/folate receptor-α (FOLR1)/mucin-1 (MUC1) using different configurations of capture and detection biomarkers. MUC1 capture exhibited the greatest separation and strongest signal among the three configurations tested.

The authors hypothesized that the colocalization of multiple cancer-associated biomarkers could improve the specificity for distinguishing dilute cancer EVs from normal EVs. To test this, the authors spiked plasma from healthy control subjects with EVs derived from ovarian cancer cell lines and evaluated discrimination performance using combinations of one, two, and three unique biomarkers. Spiked plasma was prepared by mixing purified COV413A EVs with a female plasma pool derived from 90 healthy women at a final concentration of 1 × 10^8^ COV413A particles/mL. Unspiked and spiked plasma pools were characterized in the assay using biomarker combinations containing BST2, FOLR1, and/or MUC1 in various capture/detection assay configurations. Single-biomarker combinations comprising capture and detection antibodies targeting MUC1 only (MUC1 capture, MUC1 + MUC1 detection) (Figure 4D), BST2 only, or FOLR1 only (data not shown), generally exhibited poor discrimination between unspiked and spiked plasma samples (median ΔC_T_ = 0.2). However, discrimination performance improved in some cases for biomarker combinations requiring the colocalization of two unique cancer-associated biomarkers in a biomarker-dependent manner (Figure 4D). For example, MUC1 capture/FOLR1 + FOLR1 capture resulted in poor discrimination of unspiked versus spiked plasma (ΔC_T_ = 0.6), whereas MUC1 capture/BST2 + BST2 detection resulted in improved discrimination (ΔC_T_ = 3.2). Assay configurations targeting all three biomarkers generally exhibited superior discrimination, which varied on the basis of the biomarker selected for immunoaffinity capture (ΔC_T_ = 3.1 to 6.1) (Supplemental Figure S5). Among the three triplet configurations tested, MUC1 capture/FOLR1 + BST2 detection exhibited the greatest separation of unspiked versus spiked plasma (ΔC_T_ = 6.1) (Figure 4D). This three-biomarker combination exhibited an 18 C_T_ (approximately 260,000-fold) reduction of unspiked healthy background signal relative to the single biomarker combination MUC1 capture, MUC1 + MUC1 detection, enabling the detection of dilute cancer-derived EVs.

### Biomarkers Are Colocalized on Individual EVs from Human Cell Lines

**Figure 5:**
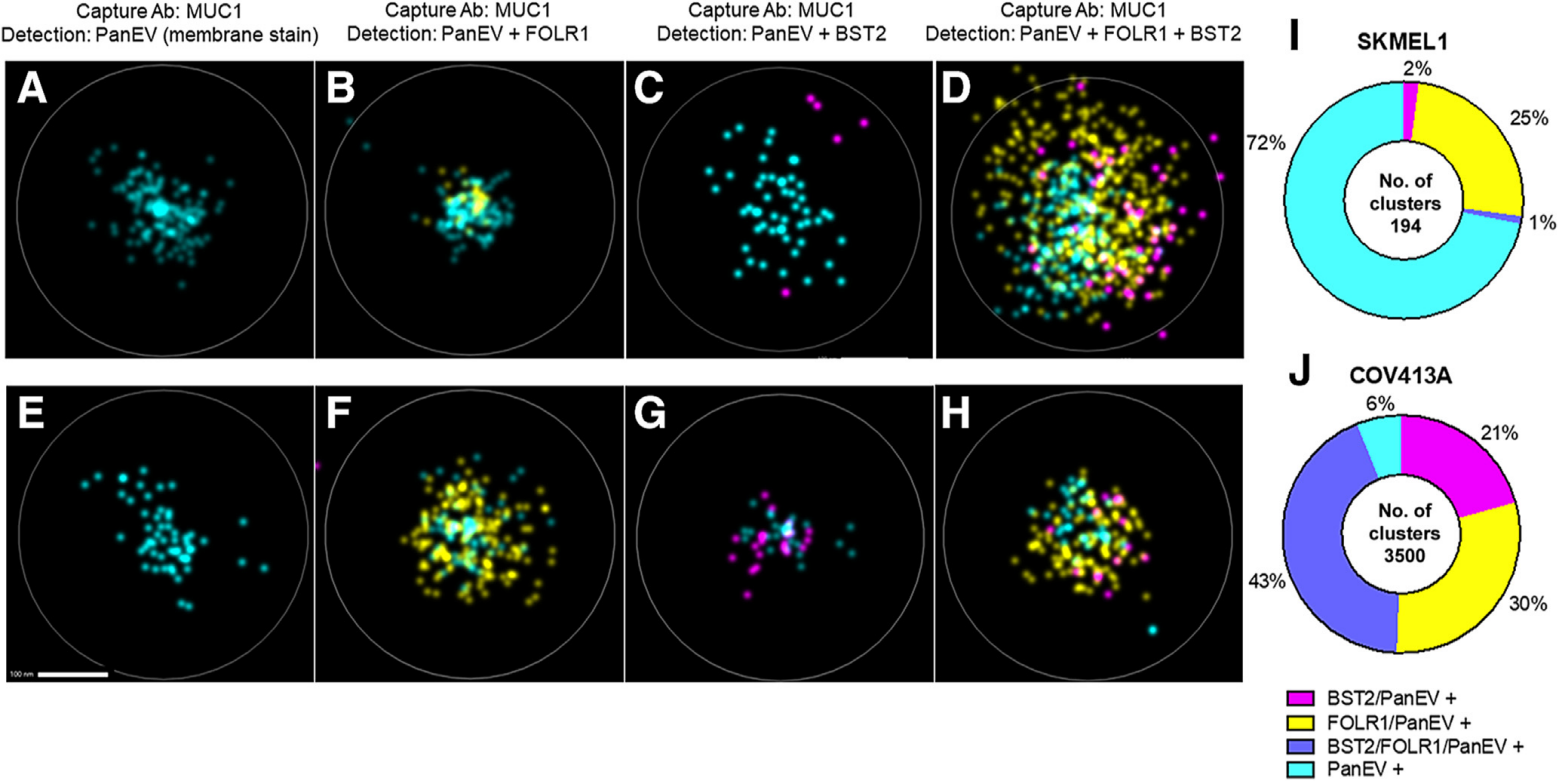
Colocalization of bone marrow stromal antigen-2 (BST2), folate receptor-α (FOLR1), and mucin-1 (MUC1) on single EVs using superresolution microscopy. EVs derived from SKMEL1 (BST2^low^/FOLR1^low^/MUC1^low^; **A**–**D**) and COV413A (BST2^high^/FOLR1^high^/MUC1^high^; **E**–**H**) cells were captured on an EV Profiler microfluidic slide (ONI) coated with a monoclonal antibody (Ab) to MUC1 (MUC1 capture Ab) before staining with a pan-EV membrane dye (PanEV) and Alexa Fluor antibody conjugates to FOLR1 (yellow; **B** and **D**), BST2 (magenta; **C** and **G**), or both (**D** and **H**). EVs were identified using AutoEV software (ONI) and verified manually. Single, double, and triple positive EVs were quantified by cluster analysis and presented for both SK-MEL-1 (**I**) and COV413A (**J**). Experimental details, imaging parameters, and software setting are described in Materials and Methods. Representative images are shown. Scale bar = 100 nm (**A**–**H**).

**Supplemental Figure S6:**
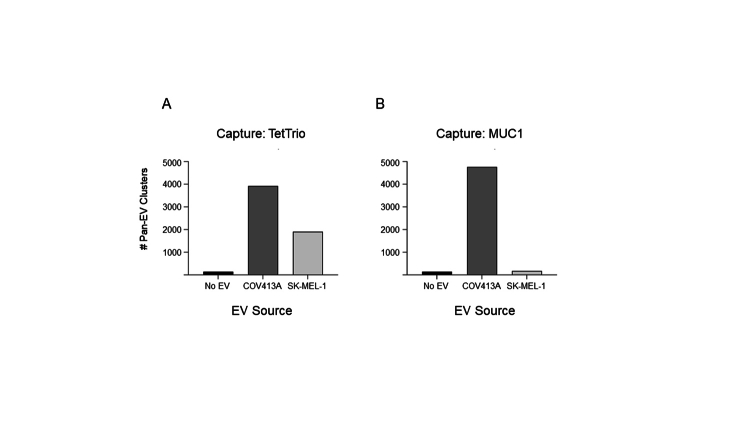
EV capture efficiency in COV413A and SK-MEL-1 cell line EVs. A total of 4 × 10^10^ EVs from COV413A [mucin-1 (MUC1)^high^/folate receptor-α (FOLR1)^high^/bone marrow stromal antigen-2 (BST2)^high^] or SK-MEL-1 (MUC1^low^/FOLR1^low^/BST2^low^) were captured on EV Profiler 2 microfluidic chips (ONI) using a cocktail of antibodies (Abs) to CD9, CD63 and CD81 (TetTrio; **A**) or MUC1 (**B**). Captured EVs were detected using a Pan-EV membrane dye using an ONI Nanoimager (software version 1.19.15.20240417195007) and ONI AutoEV (beta software version) to detect Pan-EV signal clusters, as described in Materials and Methods. No EV controls were used to establish non-specific fluorescence background signal.

To verify the extent of biomarker colocalization on individual EVs, superresolution microscopy was used to capture and identify single, double, and triple biomarker positive EVs derived from BST2^low^/FOLR1^low^/MUC1^low^ SK-MEL-1 cells (Figure 5, A–D and I) and BST2^high^/FOLR1^high^/MUC1^high^ COV413A cells (Figure 5, E–H and J). Cell line EVs captured on EV Profiler (ONI) microfluidic chips coated with antibodies against MUC1 were stained with a fluorescent membrane dye (PanEV) (Figure 5, A and E) and fluorescently conjugated antibodies to FOLR1 (Figure 5, B and F), BST2 (Figure 5, C and G), or both (Figure 5, D and H). In both cell lines, it was possible to identify single (MUC1), double (MUC1 + FOLR1 or MUC1 + BST2), and triple biomarker (MUC1 + FOLR1 + BST2) positive EVs, but the total number of EVs captured and detected from COV413A cells was significantly higher than those from SK-MEL-1 cells. In the experiment highlighted in Figure 5, there were 3500 positive clusters (EVs) identified by the CODI software in COV413A cells versus only 194 for SK-MEL-1. Representative images of individual MUC1 (MUC1 capture; PanEV detection), MUC1 + FOLR1 (MUC1 capture; PanEV + FOLR1 detection), MUC1 + BST2 (MUC1 capture; PanEV + BST2 detection), and MUC1 + FOLR1 + BST2 (MUC1 capture; PanEV + FOLR + BST2 detection) positive EVs are shown in Figure 5, A–D (SK-MEL-1) and E and F (COV413A). Notably, far fewer EVs were captured from SK-MEL-1 (MUC1^low^) than COV413A (MUC1^high^) using MUC1 capture (Figure 5, I and J, and Supplemental Figure S6), despite a similar number of EVs used for each cell line. Capture of cell line EVs with tetraspanin antibodies to CD9, CD63, and CD81 (TetTrio) demonstrates positive EV signal, as indicated by PanEV staining in both COV413A and SK-MEL-1 relative to the MUC1 capture using similar numbers of input EVs from both cell lines (Supplemental Figure S6).

### Assay Performance Depends on Intact EVs

**Supplemental Figure S7:**
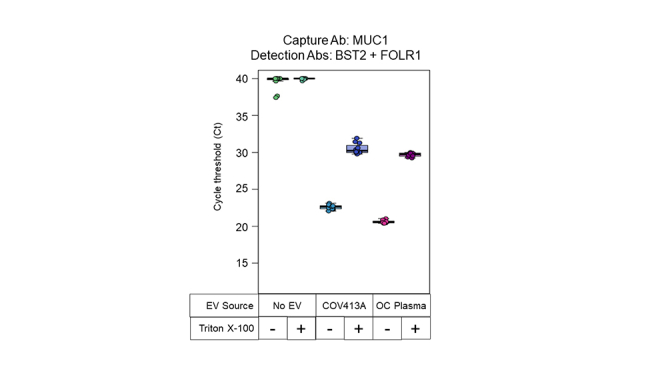
Detergent sensitivity of assay performance. Buffer alone (No EV), EVs from COV413A, or pooled human plasma from late-stage high-grade serous ovarian cancer patients' plasma (OC Plasma) were assessed using mucin-1 (MUC1) capture/folate receptor-α (FOLR1) + bone marrow stromal antigen-2 (BST2) detection antibody combination as indicated. After capture on MUC1 antibody-bead conjugates, bead-bound EVs were incubated in buffer alone (–) or buffer containing 0.075% Triton X-100 (+) to disrupt the EV membranes, as described in Materials and Methods. After a wash step to remove detergent, intact EVs were detected with anti-BST2 and anti-FOLR1 antibodies. Note that the presence of detergent results in a significant reduction in assay signal, suggesting intact EVs are required for productive readout.

To demonstrate that assay signal is derived from colocalized biomarkers on an intact EV, SEC-enriched EVs from human cell line conditioned media (COV413A) or pooled plasma from late-stage patients with HGSOC were subjected to capture with MUC1 antibodies. Before detection, captured EVs were incubated with buffer alone (–) or buffer containing 0.075% Triton X-100 (+) sufficient to disrupt intact EVs (Supplemental Figure S7).^64^ Assay signal was generated using detection antibodies to BST2 and FOLR1. Detergent treatment resulted in a significant increase (>7) in C_T_, suggesting that >99% of the signal is derived from intact EVs that are detergent sensitive.

### Assay Signal Requires Biomarker Colocalization on the Same EV

**Supplemental Figure S8:**
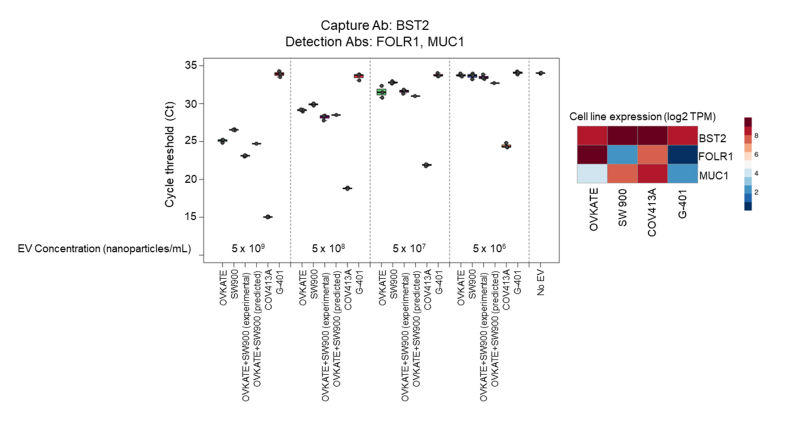
Demonstration of single EV colocalization. EVs from OVKATE [bone marrow stromal antigen-2 (BST2)^high^, folate receptor-α (FOLR)^high^, mucin-1 (MUC1)^low^], SW900 (BST2^high^, FOLR1^low^, MUC1^high^), COV413A (BST2^high^, FOLR1^high^, MUC1^high^), and G-401 (BST2^high^, FOLR1^low^, MUC1^low^) were characterized using the combination BST2 capture/FOLR1 + MUC1 detection at varying input concentrations of 5E6 to 5E9 particles/mL. EVs from OVKATE and SW900 were characterized independently or mixed 1:1. Signal from the EV mixture was compared against the linear sum of the individual EV signals to determine whether oligo-conjugated antibodies on two independent EVs can bridge to generate a PCR signal.

Theoretically, complementary DNA-conjugated antibodies located on adjacent EVs could generate a DNA template if within sufficient proximity (bridging). To assess whether bridging between EVs is observed, the authors characterized a mixture of EVs from two different cancer cell lines, OVKATE and SW900, each predicted to express high levels of only two of the three biomarkers (BST2^high^/FOLR1^high^/MUC1^low^, and BST2^high^/FOLR1^low^/MUC1^high^, respectively). The authors characterized the individual cell line EVs and a 1:1 mixture using the triplet biomarker combination BST2 capture/FOLR1 + MUC1 detection across a range of EV concentrations (5 × 10^6^ to 5 × 10^9^ particles/mL). At each concentration level, signals from the individual cell line EVs were used to calculate the expected mixture signal based on a linear sum of DNA templates. Signals from the mixtures of double-positive EVs were within 1 C_T_ of the mathematical predictions up to an input EV concentration of 5 × 10^8^ particles/mL per cell line (total EV concentration in mixture = 1 × 10^9^ particles/mL) (Supplemental Figure S8). The EV mixture containing 5 × 10^9^ particles/mL per cell line (total EV concentration = 1 × 10^10^ particles/mL) exhibited slightly stronger signal (23.1 C_T_) than mathematically predicted (24.7 C_T_), which may represent false signal from inter-EV bridging. However, this concentration represents approximately 100% of the total EV concentration in blood and is not physiologically relevant when targeting dilute cancer-associated EV populations, suggesting that the risk of inter-EV bridging leading to a false-positive signal in clinical samples is low.^38^

### Assay Linearity and Reproducibility

**Supplemental Figure S9:**
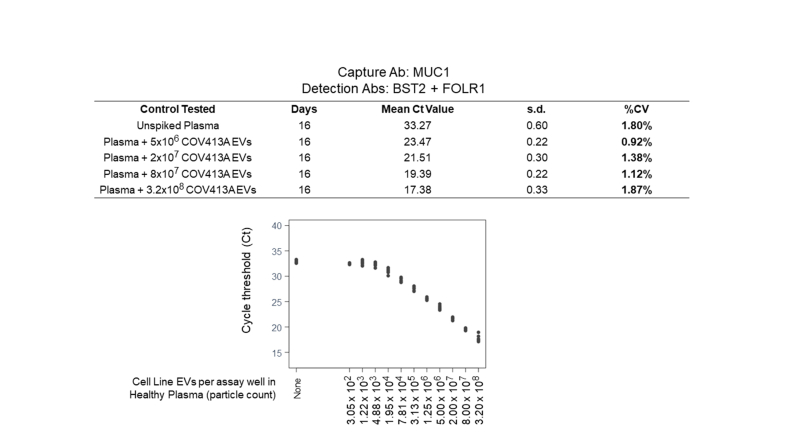
Reproducibility and linearity of the assay in cell line EV-spiked human plasma. Representative reproducibility (**top**) and linearity (**bottom**) of assay performance in COV413A EV spiked human plasma using bone marrow stromal antigen-2 (BST2)/folate receptor-α (FOLR1)/mucin-1 (MUC1). Mean C_T_ values represent the mean of duplicate assays evaluated over 16 days. Points for each spike-in represent eight technical replicates. The last dilution point in linear range was 1.95 × 10^4^, which indicates a linear range over more than 15 C_T_.

The linearity of the MUC1, BST2 + FOLR1 combination was evaluated using plasma spiked with a fourfold dilution series of EVs collected from the human ovarian cancer cell line COV413A (Supplemental Figure S9). Samples prepared in this manner were expected to cover the healthy control and ovarian cancer C_T_ range of approximately 20 to approximately 40. The dilution series was prepared four times and then each sample was run in duplicate (eight replicates) in a single assay plate. There was no high-dose hook effect observed, even at the highest spike-in level of 3.2 × 10^8^ EVs per sample well, which is well above the combination C_T_ values observed in either healthy or ovarian cancer samples. This combination was linear over the 4-log range of 19,500 to 320 million cell line EVs per well. On the basis of the results from the linearity study, five-level controls were established at 3.2 × 10^8^ EVs per well, 8 × 10^7^ EVs per well, 2 × 10^7^ EVs per well, 5 × 10^6^ EVs per well, and unspiked plasma background. Each of these full process controls was tested with the MUC1, BST2 + FOLR1 combination on 16 separate days and the between-day reproducibility of the C_T_ values was calculated from these data (Supplemental Figure S9). This combination had C_T_ interday CVs of <2% for all five-level controls.

### Assay Signal Correlates with Cell Line Gene Expression and Protein Expression

**Supplemental Figure S10:**
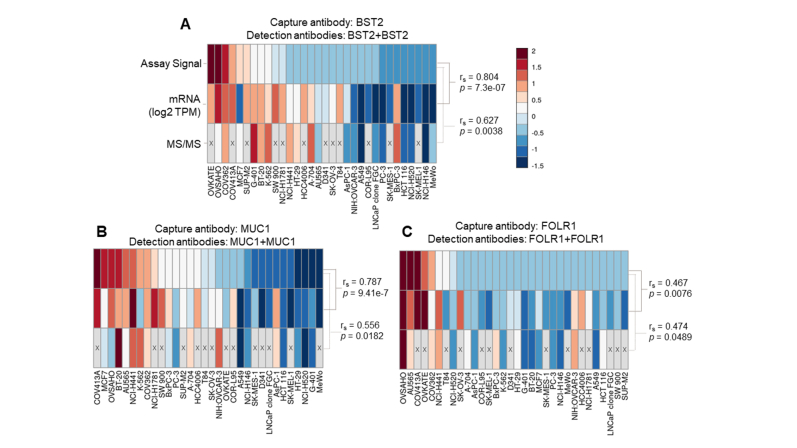
Correlation of assay signal with biomarker expression in cell line EVs. Heat maps comparing assay signal (**top row**), mRNA expression [log2 transcripts per million (TPM); **middle row**], and protein abundance, as determined by tandem mass spectroscopy (MS/MS; **bottom row**) for the biomarkers tested in this study, bone marrow stromal antigen-2 (BST2; **A**), folate receptor-α (FOLR1; **B**), and mucin-1 (MUC1; **C**). Gene expression and MS/MS data were sourced from the Cancer Cell Line Encyclopedia from the Broad Institute. The **top row** (assay signal) of each heat map is 40 minus the C_T_, the **middle row** (mRNA) is log2 TPM, and the **bottom row** (MS/MS) is the log2 ratio of tandem mass tag (TMT) reporters versus control. All data were scaled to allow comparison across data types. Spearman rank correlation (r_s_) and *P* value (*p*) for each data set are shown.

To evaluate the correlation between assay signal and cell line gene and protein expression, purified EVs from 32 human cancer cell lines were characterized at a constant nanoparticle concentration using single-biomarker capture/detect combinations directed toward BST2, FOLR1, and MUC1. Assay signal for each biomarker is summarized in Supplemental Figure S10 along with published gene expression and protein expression data (when available). Signal strength was positively correlated with gene and protein expression for all three biomarkers (Spearman correlation coefficients between 0.47 and 0.87), suggesting that cell line biomarker expression is generally predictive of EV protein composition.

### Discrimination of HGSOC in Patient Samples

**Figure 6:**
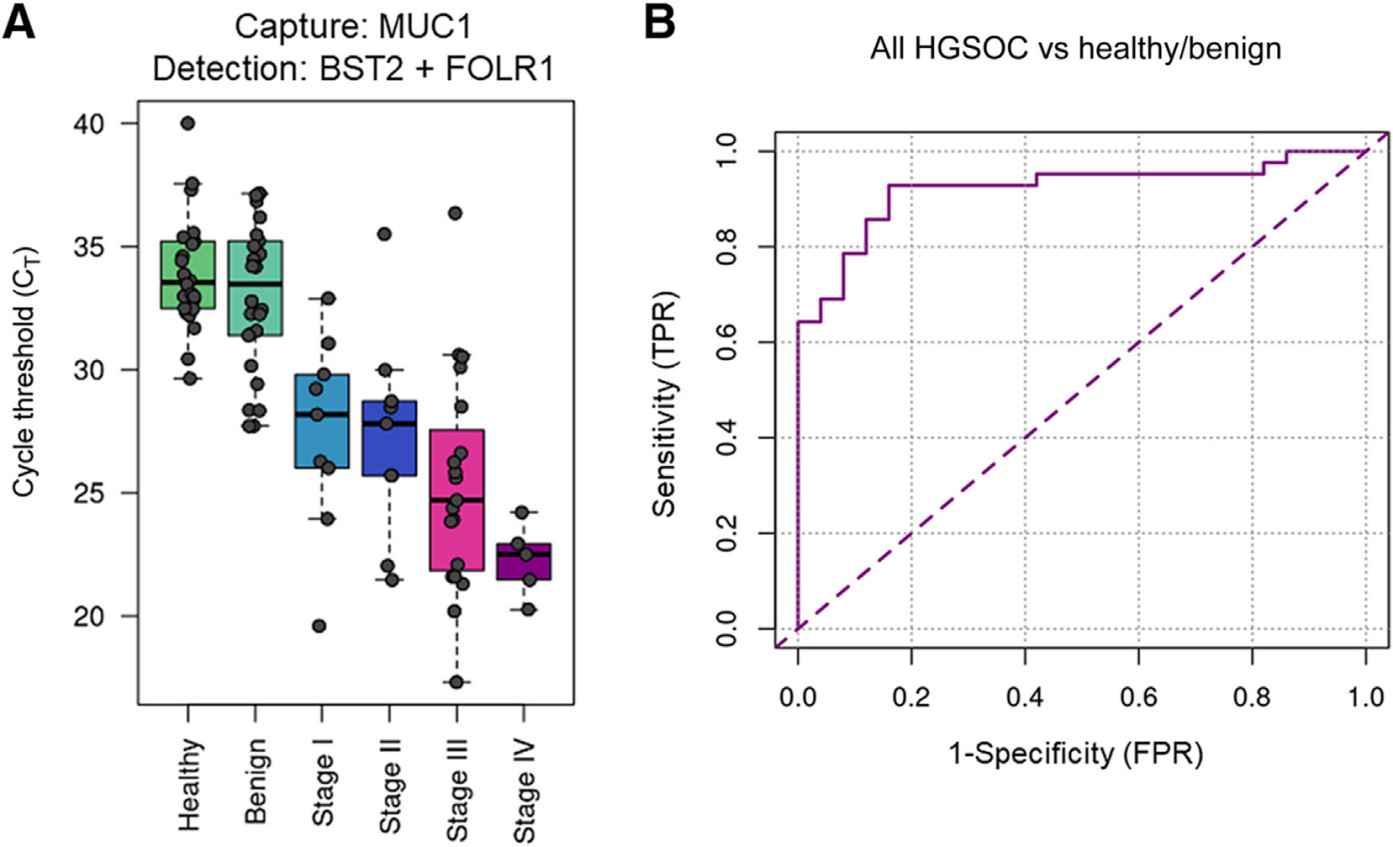
Discrimination of high-grade serous ovarian cancer (HGSOC) from healthy controls and benign ovarian masses. **A:** Performance of the mucin-1 (MUC1) capture/bone marrow stromal antigen-2 (BST2) + folate receptor-α (FOLR1) detection biomarker combination in a cohort of healthy controls, patients with benign ovarian masses, and patients with stage I, II, III, and IV HGSOC in a proof-of-concept assay. **B:** Corresponding receiver operating characteristic curve for discrimination of all HGSOC cases against healthy controls and benign adnexal masses (area under the curve = 0.920). *n* = 24 healthy controls (**A**); *n* = 26 patients with benign ovarian masses (**A**); *n* = 9 patients with stage I and II HGSOC (**A**); *n* = 15 patients with stage III HGSOC (**A**); *n* = 4 patients with stage IV HGSOC (**A**). FPR, false positive rate; TPR, true positive rate.

**Supplemental Figure S11:**
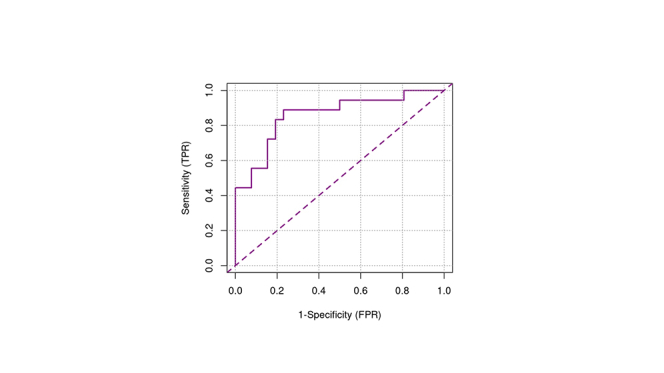
Discrimination of early-stage high-grade serous ovarian cancer (HGSOC) versus benign adnexal masses. Receiver operating characteristic curve for discrimination of stage I/II HGSOC versus benign adnexal masses using a biomarker combination targeting bone marrow stromal antigen-2/folate receptor-α/mucin-1–positive EVs (area under the curve = 0.859). *n* = 18 stage I/II HGSOC; *n* = 24 benign adnexal masses. FPR, false positive rate; TPR, true positive rate.

To assess feasibility for the detection of early-stage cancer, the authors applied the assay to a clinical cohort of 92 women composed of 42 HGSOC cases (18 stage I/II, 24 stage III/IV), 26 benign adnexal masses, and 24 healthy controls. Plasma from each woman was characterized using the BST2/FOLR1/MUC1 biomarker combination targeting BST2/FOLR1/MUC1-positive EVs. The results are summarized in Figure 6. In this limited proof-of-concept study, the biomarker combination of BST2/FOLR1/MUC1 achieved 69% sensitivity (95% CI, 52.9%–82.4%) for all stages of disease at a specificity of 96% relative to healthy controls and benign adnexal masses and an area under the curve value of 0.920 (Figure 6). Most importantly, this biomarker combination readily discriminated between early-stage HGSOC and benign disease, as demonstrated by an ROC curve area under the curve value of 0.859 (Supplemental Figure S11. This performance has been further improved in subsequently developed assay configurations,^65^ suggesting this approach may have clinical utility in detecting early-stage HGSOC and discriminating ovarian malignancy from benign adnexal masses.

## Discussion

Cancer screening has been shown to reduce cancer-associated mortality through the detection of localized disease (ie, more amenable to treatment), underlining the clinical need for more accurate and accessible screening tests.^5^ Despite considerable effort developing blood-based screening approaches, technical challenges have constrained test performance. Early-stage tumors are small, limiting the abundance of the available analytes for measurement in liquid biopsies. Cancer misregulates normal physiological processes, which means the analytes produced by cancers are often also produced by normal tissues, either in the tissue of origin or other parts of the body. Even when arising from the same tissue type, cancer utilizes blind evolutionary opportunism and therefore the mechanisms that lead to carcinogenesis can vary widely. Measuring colocalized cancer-associated biomarkers on intact EVs addresses each of these challenges. Specifically, EVs are a stochastic sampling of the cell of origin bearing cancer-associated biomarkers both on their surface and as cargo.^66^^,^^67^ They are released continuously from tumor cells because of their anti-inflammatory and tumor-supporting functions and are stable enough to tolerate processing.^38^

Here, we describe a novel, noninvasive method for the early detection of cancer through the measurement of colocalized cancer-associated biomarkers on the surface of extracellular vesicles. EVs are an emerging analyte class with features well suited to use in diagnostic testing and represent an area of science undergoing rapid advances in the understanding of basic biology as well as research and clinical applications.^32^ There are biological and technical limitations to the approach described here that are important considerations informing ongoing assay development. EVs derived from hematopoietic lineage cells comprise 99.8% of all EVs in human plasma, and thus biomarker targets that are highly expressed in blood cells may generate assay background.^35^^,^^38^^,^^67^ Assay performance is dependent on the quality and availability of affinity reagents, such as antibodies, which are compatible with the bioconjugation chemistries used in this work. Promising cancer-associated biomarkers lacking compatible affinity reagents with suitable performance characteristics may restrict potentially useful biomarker combinations from inclusion in assays. Among biomarkers with suitable affinity reagents, we observed a range in discrimination performance, potentially resulting from variability in healthy background signal. Variability in assay specificity may be driven by several factors, including biomarker expression, antibody affinity and specificity, and the orientation of the assay design (ie, the choice of biomarkers for use in immunoaffinity capture versus the detection step; Supplemental Figure S5). The studies to evaluate test performance in clinical samples provide encouraging evidence that the assay will have utility in identifying early-stage cancers and discriminating malignant from healthy samples but represent proof of concept only. Clinical samples were sourced from multiple vendors/clinical sites and included two different types of plasma. Further studies have been conducted to assess the clinical performance of the assay for the detection of HGSOC in well-powered case-control studies using clinical samples collected prospectively under standardized protocols.^65^

Measuring colocalized biomarkers on single EVs using the assay described herein is a promising approach for early-stage cancer detection. It addresses the biological abundance limitations of genomic liquid biopsy approaches while offering improved specificity over bulk EV measurements. The assay measures EV surface markers and thereby leverages the natural enrichment of membrane-bound epitopes relative to cargo proteins because of the 100-fold increase in surface area/volume ratio between an EV and its cell of origin. Moreover, it can interrogate billions of EVs simultaneously, enabling the detection of dilute (<1 in 100,000)^35^ cancer-associated EVs, reaffirming the observation that the assay can be used to detect EVs from early-stage tumors. The qPCR-based readout enhances assay technical sensitivity relative to conventional enzyme-amplified sandwich immunoassays, similar to other assays that leverage immuno-PCR,^39^^,^^68^^,^^69^ proximity ligation,^56^^,^^70^^,^^71^ and proximity extension technology.^72^^,^^73^

The small sample volume requirement, compatibility with standard blood collection tubes, and use of low-cost qPCR for signal generation are features that may help to enable broad adoption, including in lower-resource settings. Advances in affinity reagent development and EV biomarker discovery may be leveraged to improve existing assays by incorporating new reagents or designing orthogonal combinations to boost assay performance. We anticipate this assay format will be readily extensible to a broad array of clinical indications, across cancer types, and beyond cancer. For example, neuronally derived EV biomarkers have been reported to track with cognitive decline in Alzheimer disease and may represent another opportunity for diagnostic utility.^74^ EV-based measurement of tumor-derived biomarkers may be helpful in guiding clinical management through use in companion diagnostic applications. For example, folate receptor-α, one of the ovarian cancer biomarkers included in the combination described in this study, is the target of several ongoing efforts focused on the development of antibody-drug-conjugate therapeutics.^75^ A liquid biopsy assay indicative of the presence of folate receptor-α–positive tumors may be helpful in identifying patients more likely to respond to targeted antibody-drug-conjugate therapy. The EV-based assay described here has the potential for immediate clinical utility in single cancer early detection, and potentially for multicancer early detection either through the rational combination of single-cancer EV tests, or as part of an integrated multiomic approach.

## Disclosure Statement

D.P.S., L.T.B., I.O.Z., A.D.C., S.B., D.M.B., M.S.K., L.T.C., T.S.-H., G.N.B., K.S.Y., T.G., and D.R.M. are current employees of Mercy BioAnalytics Inc. E.S.W.-D. is a retired Mercy BioAnalytics employee (December 31, 2023) and is currently a paid consultant of Mercy BioAnalytics Inc. She was a full-time employee when this work was performed. K.M.B., P.A.D., J.G., D.G., B.F.H., E.K.H., C.R.S., and J.C.S. are former employees of Mercy BioAnalytics Inc., who were active employees at the time this work was performed. R.W.S. is a paid consultant of Mercy BioAnalytics. L.T.B. and D.P.S. are inventors on US patent number 11,085,089 B2, Systems, Compositions and Methods for Target Entity Detection (issued August 10, 2021). L.T.B., D.P.S., E.S.W.-D., D.G., K.M.B., E.K.H., and A.D.C. are inventors on US patent application 63/417309, Composition and Methods for Detection of Ovarian Cancer (filed October 18, 2022).

## References

[1] Siegel R.L., Miller K.D., Wagle N.S., Jemal A. (2023). Cancer statistics, 2023. CA Cancer J Clin.

[2] Mariotto A.B., Enewold L., Zhao J., Zeruto C.A., Yabroff K.R. (2020). Medical care costs associated with cancer survivorship in the United States. Cancer Epidemiol Biomarkers Prev.

[3] McGarvey N., Gitlin M., Fadli E., Chung K.C. (2022). Increased healthcare costs by later stage cancer diagnosis. BMC Health Serv Res.

[4] Sung H., Ferlay J., Siegel R.L., Laversanne M., Soerjomataram I., Jemal A., Bray F. (2021). Global cancer statistics 2020: GLOBOCAN estimates of incidence and mortality worldwide for 36 cancers in 185 countries. CA Cancer J Clin.

[5] Crosby D., Bhatia S., Brindle K.M., Coussens L.M., Dive C., Emberton M., Esener S., Fitzgerald R.C., Gambhir S.S., Kuhn P., Rebbeck T.R., Balasubramanian S. (2022). Early detection of cancer. Science.

[6] Grossman D.C., Curry S.J., Owens D.K., Barry M.J., Davidson K.W., Doubeni C.A., Epling J.W., Kemper A.R., Krist A.H., Kurth A.E., Landefeld C.S., Mangione C.M., Phipps M.G., Silverstein M., Simon M.A., Tseng C.-W., US Preventive Services Task Force (2018). Screening for ovarian cancer: US Preventive Services Task Force recommendation statement. JAMA.

[7] Grossman D.C., Curry S.J., Owens D.K., Bibbins-Domingo K., Caughey A.B., Davidson K.W., Doubeni C.A., Ebell M., Epling J.W., Kemper A.R., Krist A.H., Kubik M., Landefeld C.S., Mangione C.M., Silverstein M., Simon M.A., Siu A.L., Tseng C.-W., US Preventive Services Task Force (2018). Screening for prostate cancer: US Preventive Services Task Force recommendation statement. JAMA.

[8] Curry S.J., Krist A.H., Owens D.K., Barry M.J., Caughey A.B., Davidson K.W., Doubeni C.A., Epling J.W., Kemper A.R., Kubik M., Landefeld C.S., Mangione C.M., Phipps M.G., Silverstein M., Simon M.A., Tseng C.-W., Wong J.B., US Preventive Services Task Force (2018). Screening for cervical cancer: US Preventive Services Task Force recommendation statement. JAMA.

[9] US Preventive Services Task Force (2009). Screening for breast cancer: U.S. Preventive Services Task Force recommendation statement. Ann Intern Med.

[10] Krist A.H., Davidson K.W., Mangione C.M., Barry M.J., Cabana M., Caughey A.B., Davis E.M., Donahue K.E., Doubeni C.A., Kubik M., Landefeld C.S., Li L., Ogedegbe G., Owens D.K., Pbert L., Silverstein M., Stevermer J., Tseng C.-W., Wong J.B., US Preventive Services Task Force (2021). Screening for lung cancer: US Preventive Services Task Force recommendation statement. JAMA.

[11] Davidson K.W., Barry M.J., Mangione C.M., Cabana M., Caughey A.B., Davis E.M., Donahue K.E., Doubeni C.A., Krist A.H., Kubik M., Li L., Ogedegbe G., Owens D.K., Pbert L., Silverstein M., Stevermer J., Tseng C.-W., Wong J.B., US Preventive Services Task Force (2021). Screening for colorectal cancer: US Preventive Services Task Force recommendation statement. JAMA.

[12] Pavlik E.J., van Nagell J.R. (2013). Early detection of ovarian tumors using ultrasound. Womens Health.

[13] Pinsky P.F. (2014). Assessing the benefits and harms of low-dose computed tomography screening for lung cancer. Lung Cancer Manag.

[14] van Nagell J.R., DePriest P.D., Ueland F.R., DeSimone C.P., Cooper A.L., McDonald J.M., Pavlik E.J., Kryscio R.J. (2007). Ovarian cancer screening with annual transvaginal sonography: findings of 25,000 women screened. Cancer.

[15] Menon U., Gentry-Maharaj A., Hallett R., Ryan A., Burnell M., Sharma A., Lewis S., Davies S., Philpott S., Lopes A., Godfrey K., Oram D., Herod J., Williamson K., Seif M.W., Scott I., Mould T., Woolas R., Murdoch J., Dobbs S., Amso N.N., Leeson S., Cruickshank D., McGuire A., Campbell S., Fallowfield L., Singh N., Dawnay A., Skates S.J., Parmar M., Jacobs I. (2009). Sensitivity and specificity of multimodal and ultrasound screening for ovarian cancer, and stage distribution of detected cancers: results of the prevalence screen of the UK Collaborative Trial of Ovarian Cancer Screening (UKCTOCS). Lancet Oncol.

[16] Menon U., Gentry-Maharaj A., Burnell M., Singh N., Ryan A., Karpinskyj C., Carlino G., Taylor J., Massingham S.K., Raikou M., Kalsi J.K., Woolas R., Manchanda R., Arora R., Casey L., Dawnay A., Dobbs S., Leeson S., Mould T., Seif M.W., Sharma A., Williamson K., Liu Y., Fallowfield L., McGuire A.J., Campbell S., Skates S.J., Jacobs I.J., Parmar M. (2021). Ovarian cancer population screening and mortality after long-term follow-up in the UK Collaborative Trial of Ovarian Cancer Screening (UKCTOCS): a randomised controlled trial. Lancet.

[17] Ignatiadis M., Sledge G.W., Jeffrey S.S. (2021). Liquid biopsy enters the clinic - implementation issues and future challenges. Nat Rev Clin Oncol.

[18] Lone S.N., Nisar S., Masoodi T., Singh M., Rizwan A., Hashem S., El-Rifai W., Bedognetti D., Batra S.K., Haris M., Bhat A.A., Macha M.A. (2022). Liquid biopsy: a step closer to transform diagnosis, prognosis and future of cancer treatments. Mol Cancer.

[19] Dang D.K., Park B.H. (2022). Circulating tumor DNA: current challenges for clinical utility. J Clin Invest.

[20] Desai A., Lovly C.M. (2023). Challenges in the implementation of ultrasensitive liquid biopsy approaches in precision oncology. J Immunother Cancer.

[21] Yu D., Li Y., Wang M., Gu J., Xu W., Cai H., Fang X., Zhang X. (2022). Exosomes as a new frontier of cancer liquid biopsy. Mol Cancer.

[22] Yu W., Hurley J., Roberts D., Chakrabortty S.K., Enderle D., Noerholm M., Breakefield X.O., Skog J.K. (2021). Exosome-based liquid biopsies in cancer: opportunities and challenges. Ann Oncol.

[23] Petricoin E.F., Ardekani A.M., Hitt B.A., Levine P.J., Fusaro V.A., Steinberg S.M., Mills G.B., Simone C., Fishman D.A., Kohn E.C., Liotta L.A. (2002). Use of proteomic patterns in serum to identify ovarian cancer. Lancet.

[24] Sorace J.M., Zhan M. (2003). A data review and re-assessment of ovarian cancer serum proteomic profiling. BMC Bioinf.

[25] Diamandis E.P. (2004). Proteomic patterns to identify ovarian cancer: 3 years on. Expert Rev Mol Diagn.

[26] Cabús L., Lagarde J., Curado J., Lizano E., Pérez-Boza J. (2022). Current challenges and best practices for cell-free long RNA biomarker discovery. Biomark Res.

[27] Han H., Jiang X. (2014). Overcome support vector machine diagnosis overfitting. Cancer Inform.

[28] van Niel G., D’Angelo G., Raposo G. (2018). Shedding light on the cell biology of extracellular vesicles. Nat Rev Mol Cell Biol.

[29] Willms E., Cabañas C., Mäger I., Wood M.J.A., Vader P. (2018). Extracellular vesicle heterogeneity: subpopulations, isolation techniques, and diverse functions in cancer progression. Front Immunol.

[30] Bebelman M.P., Smit M.J., Pegtel D.M., Baglio S.R. (2018). Biogenesis and function of extracellular vesicles in cancer. Pharmacol Ther.

[31] Jerabkova-Roda K., Dupas A., Osmani N., Hyenne V., Goetz J.G. (2022). Circulating extracellular vesicles and tumor cells: sticky partners in metastasis. Trends Cancer Res.

[32] Ghodasara A., Raza A., Wolfram J., Salomon C., Popat A. (2023). Clinical translation of extracellular vesicles. Adv Healthc Mater.

[33] Amelio I., Bertolo R., Bove P., Buonomo O.C., Candi E., Chiocchi M., Cipriani C., Di Daniele N., Ganini C., Juhl H., Mauriello A., Marani C., Marshall J., Montanaro M., Palmieri G., Piacentini M., Sica G., Tesauro M., Rovella V., Tisone G., Shi Y., Wang Y., Melino G. (2020). Liquid biopsies and cancer omics. Cell Death Discov.

[34] Avanzini S., Kurtz D.M., Chabon J.J., Moding E.J., Hori S.S., Gambhir S.S., Alizadeh A.A., Diehn M., Reiter J.G. (2020). A mathematical model of ctDNA shedding predicts tumor detection size. Sci Adv.

[35] Ferguson S., Weissleder R. (2020). Modeling EV kinetics for use in early cancer detection. Adv Biosyst.

[36] Liu J., Chen Y., Pei F., Zeng C., Yao Y., Liao W., Zhao Z. (2021). Extracellular vesicles in liquid biopsies: potential for disease diagnosis. Biomed Res Int.

[37] Ferguson S., Yang K.S., Weissleder R. (2022). Single extracellular vesicle analysis for early cancer detection. Trends Mol Med.

[38] Johnsen K.B., Gudbergsson J.M., Andresen T.L., Simonsen J.B. (2019). What is the blood concentration of extracellular vesicles? implications for the use of extracellular vesicles as blood-borne biomarkers of cancer. Biochim Biophys Acta Rev Cancer.

[39] Sano T., Smith C.L., Cantor C.R. (1992). Immuno-PCR: very sensitive antigen detection by means of specific antibody-DNA conjugates. Science.

[40] Fredriksson S., Gullberg M., Jarvius J., Olsson C., Pietras K., Gústafsdóttir S.M., Ostman A., Landegren U. (2002). Protein detection using proximity-dependent DNA ligation assays. Nat Biotechnol.

[41] Tavoosidana G., Ronquist G., Darmanis S., Yan J., Carlsson L., Wu D., Conze T., Ek P., Semjonow A., Eltze E., Larsson A., Landegren U.D., Kamali-Moghaddam M. (2011). Multiple recognition assay reveals prostasomes as promising plasma biomarkers for prostate cancer. Proc Natl Acad Sci U S A.

[42] Wu D., Yan J., Shen X., Sun Y., Thulin M., Cai Y., Wik L., Shen Q., Oelrich J., Qian X., Dubois K.L., Ronquist K.G., Nilsson M., Landegren U., Kamali-Moghaddam M. (2019). Profiling surface proteins on individual exosomes using a proximity barcoding assay. Nat Commun.

[43] Koshiyama M., Matsumura N., Konishi I. (2017). Subtypes of ovarian cancer and ovarian cancer screening. Diagnostics.

[44] Wiener J., Kokotek D., Rosowski S., Lickert H., Meier M. (2020). Preparation of single- and double-oligonucleotide antibody conjugates and their application for protein analytics. Sci Rep.

[45] Yang L.-Q., Hu H.-Y., Han Y., Tang Z.-Y., Gao J., Zhou Q.-Y., Liu Y.-X., Chen H.-S., Xu T.-N., Ao L., Xu Y., Che X., Jiang Y.-B., Xu C.-W., Zhang X.-C., Jiang Y.-X., Heger M., Wang X.-M., Cheng S.-Q., Pan W.-W. (2022). CpG-binding protein CFP1 promotes ovarian cancer cell proliferation by regulating BST2 transcription. Cancer Gene Ther.

[46] Januchowski R., Sterzyńska K., Zawierucha P., Ruciński M., Świerczewska M., Partyka M., Bednarek-Rajewska K., Brązert M., Nowicki M., Zabel M., Klejewski A. (2017). Microarray-based detection and expression analysis of new genes associated with drug resistance in ovarian cancer cell lines. Oncotarget.

[47] Varaganti P., Buddolla V., Lakshmi B.A., Kim Y.-J. (2023). Recent advances in using folate receptor 1 (FOLR1) for cancer diagnosis and treatment, with an emphasis on cancers that affect women. Life Sci.

[48] Giampaolino P., Foreste V., Della Corte L., Di Filippo C., Iorio G., Bifulco G. (2020). Role of biomarkers for early detection of ovarian cancer recurrence. Gland Surg.

[49] Bax H.J., Chauhan J., Stavraka C., Santaolalla A., Osborn G., Khiabany A. (2023). Folate receptor alpha in ovarian cancer tissue and patient serum is associated with disease burden and treatment outcomes. Br J Cancer.

[50] Jain S., Nadeem N., Ulfenborg B., Mäkelä M., Ruma S.A., Terävä J., Huhtinen K., Leivo J., Kristjansdottir B., Pettersson K., Sundfeldt K., Gidwani K. (2022). Diagnostic potential of nanoparticle aided assays for MUC16 and MUC1 glycovariants in ovarian cancer. Int J Cancer.

[51] Gautam S.K., Khan P., Natarajan G., Atri P., Aithal A., Ganti A.K., Batra S.K., Nasser M.W., Jain M. (2023). Mucins as potential biomarkers for early detection of cancer. Cancers.

[52] Kufe D.W. (2009). Mucins in cancer: function, prognosis and therapy. Nat Rev Cancer.

[53] Trinidad C., Pathak H., Cheng S., Tzeng S.-C., Madan R., Sardiu M., Bantis L., Deighan C., Jewell A., Zeng Y., Godwin A. (2023). Lineage specific extracellular vesicle-associated protein biomarkers for the early detection of high grade serous ovarian cancer. Sci Rep.

[54] Yu H., Bian Q., Wang X., Wang X., Lai L., Wu Z., Zhao Z., Ban B. (2024). Bone marrow stromal cell antigen 2: tumor biology, signaling pathway and therapeutic targeting (review). Oncol Rep.

[55] Walter-Yohrling J., Cao X., Callahan M., Weber W., Morgenbesser S., Madden S.L., Wang C., Teicher B.A. (2003). Identification of genes expressed in malignant cells that promote invasion. Cancer Res.

[56] Giampaolino P., Della Corte L., Foreste V., Vitale S.G., Chiofalo B., Cianci S., Zullo F., Bifulco G. (2019). Unraveling a difficult diagnosis: the tricks for early recognition of ovarian cancer. Minerva Med.

[57] Gonzalez T., Muminovic M., Nano O., Vulfovich M. (2024). Folate receptor alpha-a novel approach to cancer therapy. Int J Mol Sci.

[58] Barani M., Bilal M., Sabir F., Rahdar A., Kyzas G.Z. (2021). Nanotechnology in ovarian cancer: diagnosis and treatment. Life Sci.

[59] Cox K.E., Liu S., Lwin T.M., Hoffman R.M., Batra S.K., Bouvet M. (2023). The mucin family of proteins: candidates as potential biomarkers for colon cancer. Cancers.

[60] Théry C., Witwer K.W., Aikawa E., Alcaraz M.J., Anderson J.D., Andriantsitohaina R., Antoniou A., Arab T., Archer F. (2018). Minimal information for studies of extracellular vesicles 2018 (MISEV2018): a position statement of the International Society for Extracellular Vesicles and update of the MISEV2014 guidelines. J Extracell Vesicles.

[61] Lobb R.J., Becker M., Wen S.W., Wong C.S.F., Wiegmans A.P., Leimgruber A., Möller A. (2015). Optimized exosome isolation protocol for cell culture supernatant and human plasma. J Extracell Vesicles.

[62] Veerman R.E., Teeuwen L., Czarnewski P., Güclüler Akpinar G., Sandberg A., Cao X., Pernemalm M., Orre L.M., Gabrielsson S., Eldh M. (2021). Molecular evaluation of five different isolation methods for extracellular vesicles reveals different clinical applicability and subcellular origin. J Extracell Vesicles.

[63] Cizmar P., Yuana Y. (2017). Detection and characterization of extracellular vesicles by transmission and cryo-transmission electron microscopy. Methods Mol Biol.

[64] Osteikoetxea X., Sódar B., Németh A., Szabó-Taylor K., Pálóczi K., Vukman K.V., Tamási V., Balogh A., Kittel Á., Pállinger É., Buzás E.I. (2015). Differential detergent sensitivity of extracellular vesicle subpopulations. Org Biomol Chem.

[65] Winn-Deen E.S., Bortolin L.T., Gusenleitner D., Biette K.M., Copeland K., Gentry-Maharaj A., Apostolidou S., Couvillon A.D., Salem D.P., Banerjee S., Grosha J., Zabroski I.O., Sedlak C.R., Byrne D.M., Hamzeh B.F., King M.S., Cuoco L.T., Duff P.A., Manning B.J., Hawkins T.B., Mattoon D., Guettouche T., Skates S.J., Jamieson A., McAlpine J.N., Huntsman D., Menon U. (2024). Improving specificity for ovarian cancer screening using a novel extracellular vesicle–based blood test: performance in a training and verification cohort. J Mol Diagn.

[66] Doyle L.M., Wang M.Z. (2019). Overview of extracellular vesicles, their origin, composition, purpose, and methods for exosome isolation and analysis. Cells.

[67] Li Y., He X., Li Q., Lai H., Zhang H., Hu Z., Li Y., Huang S. (2020). EV-origin: enumerating the tissue-cellular origin of circulating extracellular vesicles using exLR profile. Comput Struct Biotechnol J.

[68] Niemeyer C.M., Adler M., Wacker R. (2005). Immuno-PCR: high sensitivity detection of proteins by nucleic acid amplification. Trends Biotechnol.

[69] Stiller C., Viktorsson K., Paz Gomero E., Hååg P., Arapi V., Kaminskyy V.O., Kamali C., De Petris L., Ekman S., Lewensohn R., Karlström A.E. (2021). Detection of tumor-associated membrane receptors on extracellular vesicles from non-small cell lung cancer patients via immuno-PCR. Cancers.

[70] Weibrecht I., Leuchowius K.-J., Clausson C.-M., Conze T., Jarvius M., Howell W.M., Kamali-Moghaddam M., Söderberg O. (2010). Proximity ligation assays: a recent addition to the proteomics toolbox. Expert Rev Proteomics.

[71] Manouchehri Doulabi E., Fredolini C., Gallini R., Löf L., Shen Q., Ikebuchi R., Dubois L., Azimi A., Loudig O., Gabrielsson S., Landegren U., Larsson A., Bergquist J., Kamali-Moghaddam M. (2022). Surface protein profiling of prostate-derived extracellular vesicles by mass spectrometry and proximity assays. Commun Biol.

[72] Larssen P., Wik L., Czarnewski P., Eldh M., Löf L., Ronquist K.G., Dubois L., Freyhult E., Gallant C.J., Oelrich J., Larsson A., Ronquist G., Villablanca E.J., Landegren U., Gabrielsson S., Kamali-Moghaddam M. (2017). Tracing cellular origin of human exosomes using multiplex proximity extension assays. Mol Cell Proteomics.

[73] Lundberg M., Eriksson A., Tran B., Assarsson E., Fredriksson S. (2011). Homogeneous antibody-based proximity extension assays provide sensitive and specific detection of low-abundant proteins in human blood. Nucleic Acids Res.

[74] Eren E., Leoutsakos J.-M., Troncoso J., Lyketsos C.G., Oh E.S., Kapogiannis D. (2022). Neuronal-derived EV biomarkers track cognitive decline in alzheimer’s disease. Cells.

[75] El Bairi K., Al Jarroudi O., Afqir S. (2021). Revisiting antibody-drug conjugates and their predictive biomarkers in platinum-resistant ovarian cancer. Semin Cancer Biol.

