# Supplementary material for: Colocalization of Cancer-Associated Biomarkers on Single Extracellular Vesicles for Early Detection of Cancer
Source: J Mol Diagn. 2024 Sep 24;26(12):1109–28. doi: 10.1016/j.jmoldx.2024.08.006 (PMC13169315; doi:10.1016/j.jmoldx.2024.08.006)
Supplement: Supplementary Table S1 [file mmc1.docx]

**Supplemental Table 1: Human cancer cell lines used in this study.**

| **Cell Line Name** | **Vendor** | **Catalog Number** | **RRID** |
| --- | --- | --- | --- |
| A549 | ATCC | CCL-185 | CVCL_0023 |
| A704 | ATCC | HTB-45 | CVCL_1065 |
| AsPC1 | ATCC | CRL-1682 | CVCL_0152 |
| AU565 | ATCC | CRL-2351 | CVCL_1074 |
| BT-20 | ATCC | HTB-19 | CVCL_0178 |
| BxPC3 | ATCC | CRL-1687 | CVCL_0186 |
| COR-L-95 | Sigma-Aldrich/ECACC | 96020733 | CVCL_2418 |
| COV362 | Sigma-Aldrich/ECACC | 07071910 | CVCL_2420 |
| COV413A | Sigma-Aldrich/ECACC | 07071905 | CVCL_2422 |
| D341 | ATCC | HTB-187 | CVCL_0018 |
| G-401 | ATCC | CRL-1441 | CVCL_0270 |
| HCC4006 | ATCC | CRL-2871 | CVCL_1269 |
| HCT-116 | ATCC | CCL-247 | CVCL_0291 |
| HT-29 | ATCC | HTB-38 | CVCL_0320 |
| K-562 | ATCC | CCL-243 | CVCL_0004 |
| LnCaP (clone FGC) | ATCC | CRL-1740 | CVCL_1379 |
| MCF7 | ATCC | HTB-22 | CVCL_0031 |
| MeWo | ATCC | HTB-65 | CVCL_0445 |
| NCI-H146 | ATCC | HTB-173 | CVCL_1473 |
| NCI-H1781 | ATCC | CRL-5894 | CVCL_1494 |
| NCI-H441 | ATCC | HTB-174 | CVCL_1561 |
| NCI-H520 | ATCC | HTB-182 | CVCL_1566 |
| OVCAR3 | ATCC | HTB-161 | CVCL_0465 |
| OVISE | JCRB | JCRB1043 | CVCL_3116 |
| OVKATE | JCRB | JCRB1044 | CVCL_3110 |
| OVSAHO | JCRB | JCRB1046 | CVCL_3114 |
| PC-3 | ATCC | CRL-1345 | CVCL_0035 |
| SK-MEL-1 | ATCC | HTB-67 | CVCL_0068 |
| SK-MES-1 | ATCC | HTB-58 | CVCL_0630 |
| SK-OV-3 | ATCC | HTB-77 | CVCL_0532 |
| SUP-M2 | DSMZ | ACC 509 | CVCL_2209 |
| SW900 | ATCC | HTB-59 | CVCL_1731 |
| T47D | ATCC | HTB-133 | CVCL_0553 |
| T84 | ATCC | CCL-248 | CVCL_0555 |

Vendor Abbreviations: ATCC, American Type Culture Collection; ECACC, European Collection of Authenticated Cell Cultures; JCRB, Japanese Collection of Research Bioresources; DSMZ, Deutsche Sammlung von Mikroorganismen und Zellkulturen (German Collection of Microorganisms and Cell Cultures).
